# Sesamin Induces MCL-1-Dependent Apoptosis in Activated T Cells and Ameliorates Experimental Atopic Dermatitis

**DOI:** 10.7150/ijbs.116753

**Published:** 2025-07-24

**Authors:** Hee-Suk Park, Woo Jung Sung, Yoon-Yub Park, Jaewoo Hong, Hoon-Kyu Oh, Hyun-Su Lee

**Affiliations:** 1 Department of Physiology, Daegu Catholic University School of Medicine, Duryugongwon-ro 17-gil 33, Daegu, Korea 42472.; 2 Department of Pathology, Daegu Catholic University School of Medicine, Duryugongwon-ro 17-gil 33, Daegu, Korea 42472.

**Keywords:** Sesamin, MCL-1, apoptosis, activated T cells, atopic dermatitis, immunomodulation, T cell regulation

## Abstract

Sesamin, a natural lignan derived from *Sesamum indicum*, has been reported to possess anti-inflammatory and pro-apoptotic properties. However, its effect on T cell-mediated diseases and the underlying molecular mechanisms remain unclear. In this study, we demonstrate that sesamin selectively induces apoptosis in activated T cells through direct interaction with MCL-1, a critical anti-apoptotic protein of the Bcl-2 family. Sesamin suppressed IL-2 expression, CD69 upregulation, and proliferation in activated human and murine T cells. Molecular docking predicted strong binding of sesamin to the BH3-binding groove of MCL-1, which was validated by pull-down and co-immunoprecipitation assays. Sesamin inhibited MCL-1 phosphorylation at Ser64 and disrupted its heterodimerization with Bak, promoting caspase-3/8 cleavage and apoptotic death selectively in activated, but not resting, T cells. In a murine model of atopic dermatitis, oral administration of sesamin ameliorated pathological skin symptoms, reduced Th2/Th17 cytokine expression, serum IgE, mast cell infiltration, and lymph node hypertrophy. These effects correlated with suppressed MCL-1 activity and enhanced apoptosis in inflamed tissue. Our findings suggest that sesamin modulates immune responses via a novel MCL-1-dependent mechanism and represents a promising dietary-derived therapeutic strategy for T cell-driven chronic inflammatory diseases.

## Introduction

T cell activation is a cornerstone of adaptive immunity, yet its dysregulation contributes to the pathogenesis of numerous chronic inflammatory diseases 1. Under physiological conditions, activated effector T cells are eliminated via activation-induced cell death (AICD), a critical negative feedback mechanism that ensures immune resolution after antigen clearance 2,3. AICD primarily occurs through the intrinsic apoptotic pathway and plays a vital role in preventing chronic tissue inflammation and autoimmunity. When AICD is impaired, activated T cells persist in inflamed tissues and contribute to sustained cytokine production, aberrant tissue remodeling, and disease progression 4,5.

A key regulator of AICD is myeloid cell leukemia 1 (MCL-1), an anti-apoptotic member of the Bcl-2 protein family. MCL-1 stabilizes activated T cells by sequestering pro-apoptotic partners such as Bak and Bax, thereby preventing mitochondrial-mediated apoptosis MCL-1 6,7. Persistent antigenic stimulation in chronic inflammatory settings, leads to the continuous production of cytokines including IL-2, IL-7, IL-15, and IL-21, which in turn activate key transcriptional regulators such as STAT5, STAT3 and NFκB 8,9. These pathways enhance MCL-1 transcription and protein stability, promoting T cell survival and impairing AICD 10,11. This dysregulation contributes to the accumulation of pathogenic effector T cells and the persistence of chronic inflammation.

Atopic dermatitis (AD) is a chronic inflammatory skin disorder characterized by hyperactivation of the immune system and infiltration of effector T cells, particularly Th2 and Th17 subsets. These T cell populations contribute to AD pathology by producing pro-inflammatory cytokines such as IL-4, IL-13, and IL-17A, which promote barrier dysfunction, keratinocyte activation, and recruitment of additional immune cells 12,13. Importantly, the persistence of Th2/Th17 cells in chronic AD lesions is associated with resistance to AICD, a regulatory mechanism that normally limits effector T cell survival after antigen stimulation 14. This apoptotic resistance has been linked to elevated expression of the anti-apoptotic protein MCL-1, which inhibits mitochondrial-mediated apoptosis by neutralizing pro-apoptotic molecules such as Bak and Bim 6,13. Thus targeting MCL-1 may provide a promising strategy to restore immune balance in AD by promoting apoptosis of pathogenic T cells.

**Figure 1:**
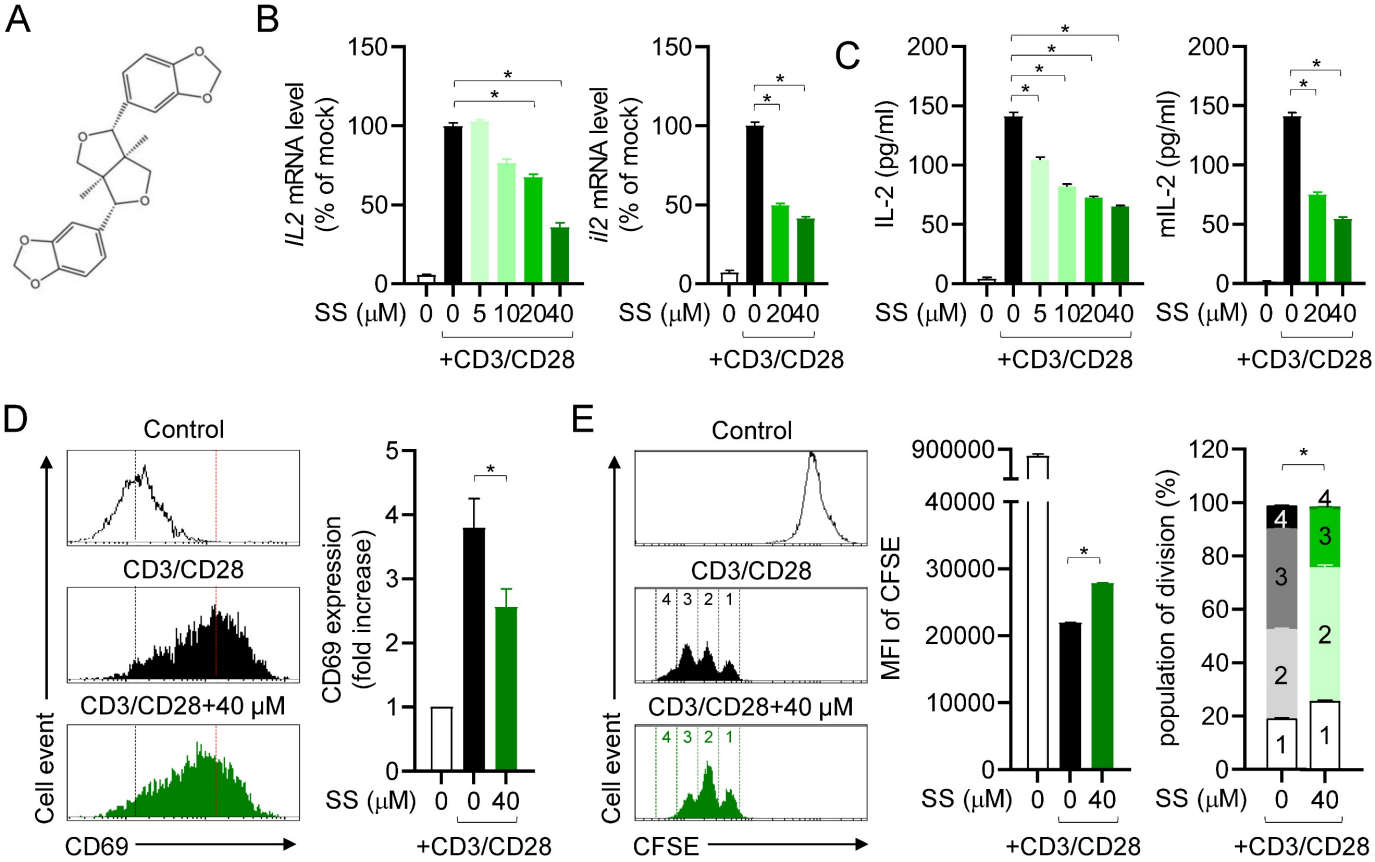
** Sesamin suppresses T cell activation marker and cytokine production.** (A) The structure of sesamin. (B) Jurkat T cells (5 X 10^5^/well, 12-well plate) or mouse CD4^+^ T cells (3 X 10^6^/well, 12-well plate) pre-treated with indicated concentration of sesamin for 1 h were stimulated with immobilized anti-CD3 (10 μg/ml) and soluble anti-CD28 (2 μg/ml) antibodies for 6 h. Harvested cells were lysed for isolation of total RNA. The mRNA level of IL-2 gene was determined by qPCR analysis. The expression of IL-2 was normalized with the expression of GAPDH. (C) Jurkat T cells (1 X 10^4^/well, 96-well plate) or mouse CD4^+^ T cells (5 X 10^4^/well, 96-well plate) pre-treated with indicated concentration of sesamin for 1 h were stimulated with immobilized anti-CD3 (10 μg/ml) and soluble anti-CD28 (2 μg/ml) antibodies for 24 h. The amount of released IL-2 was evaluated by ELISA from harvested supernatants. (D) Jurkat T cells (5 X 10^5^/well, 12-well plate) pre-treated with 40 μM sesamin for 1 h were stimulated with immobilized anti-CD3 (10 μg/ml) and soluble anti-CD28 (2 μg/ml) antibodies for 16 h. Collected cells were stained with anti-CD69 antibodies conjugated with FITC and mean fluorescence intensity was measured by flow cytometry. The mean fluorescence intensity of control group was considered as 1X and fold increase was presented compared to control group. (E) 0.5 μM CFSE-stained mouse CD4^+^ T cells (1 X 10^6^/well, 96-well plate) pre-treated with 40 μM sesamin for 1 h were stimulated with immobilized anti-CD3 (10 μg/ml) and soluble anti-CD28 (2 μg/ml) antibodies for 72 h. Harvested cells were acquired by flow cytometry to obtain CFSE fluorescence. Mean fluorescence intensity and population of each division were presented. The concentration used in (D) and (E) was selected based on prior dose-response analysis (see Methods). All results are expressed as mean ± SEM of three independent experiments. Statistical comparisons among groups were performed using one-way ANOVA with Tukey's post hoc test. A p-value less than 0.05 was considered statistically significant (*, P < 0.05).

Sesamin (C_20_H_18_O_6_; Fig. 1A), a lignan abundantly found in sesame seeds (*Sesamum indicum*), has shown anti-inflammatory and pro-apoptotic effects in various preclinical models 15,16. Despite these benefits, its mechanistic role in modulating T cell function remains poorly understood. In this study, we identify MCL-1 as a direct target of sesamin and demonstrate that sesamin induces selective apoptosis of activated T cells by disrupting MCL-1-Bak interactions, thereby promoting AICD. This mechanism results in suppressed cytokine production and resolution of inflammation in a murine model of AD. Our findings offer novel mechanistic insight into the immune-regulatory function of sesamin and suggest its potential as a natural therapeutic agent for chronic T cell-driven inflammatory disorders.

## Material and Methods

### Cell culture

Jurkat T cells (KCLB number: 40152, clone E6-1) were purchased from the Korean Cell Line Bank (KCLB, Seoul, Korea) and mouse CD4^+^ T cells were isolated from lymph nodes and spleen of Eight-week-old female BALB/c mice by using MojoSort™ magnetic cell separation system (Biolegend, San Diego, CA, USA). Jurkat T cells were cultured in RPMI medium (Welgene, Gyeongsan, Korea) supplemented with 10% fetal bovine serum (FBS, Welgene, Gyeongsan, Korea), 1 X penicillin-streptomycin (Welgene Gyeongsan, Korea), 2 mM l-glutamine (Welgene Gyeongsan, Korea). For mouse CD4^+^ T cells culture, 1 X sodium pyruvate and 50 μM 2-mercaptoethanol were additionally added to medium for Jurkat T cells. All cells were grown at 37 °C in a humidified incubator containing 5% CO_2_ and 95% air. For Jurkat T cells, 8 to 10-passage cells were used to perform the all experiments for enhancing reproductivity.

### Mice

Eight-week-old female BALB/c mice were purchased from Samtako and housed in specific pathogen-free (SPF) conditions. All animal care and experimental procedures were approved by the Animal Care and Use Committee of the Daegu Catholic University School of Medicine (approval number: DCIAFCR-230823-20-Y). All animal experiments were conducted in accordance with institutional ethical guidelines, and the reporting of procedures followed the principles of transparency and reproducibility in line with the ARRIVE guidelines.

### Sesamin preparation and use

*In vitro*, sesamin (purity ≥ 98%) obtained from ChemFaces (Wuhan, China) was dissolved in dimethyl sulfoxide (DMSO) to prepare a 100 mM stock solution, which was aliquoted and stored at -20 °C. Prior to use, the stock was diluted in culture medium. For mechanistic studies including MCL-1 phosphorylation and apoptosis assays, sesamin was tested at multiple concentrations (10, 20 and 40 µM) to assess dose-dependent effects. In contrast, functional assays such as ELISA, CFSE-based proliferation, and CD69 expression were performed using a representative concentration of 40 µM, selected based on preliminary titration experiments showing consistent and robust immunomodulatory effects. All treatments included DMSO at a final concentration of 0.1% as vehicle control to confirm that the solvent itself did not influence T cell viability or activation. For *in vivo* experiments, sesamin was suspended in 0.5% carboxymethylcellulose (CMC) and administered via oral gavage at 10 or 25 mg/kg. Oral administration of sesamin was performed 5 times in a week and repeated for 4 weeks. This dose was selected based on prior studies demonstrating both efficacy and safety in murine models of inflammation 17. Mice in the vehicle control group received the same volume of CMC without sesamin. CMC alone was used as the vehicle control and did not elicit any observable effects in control and AD mice group.

### Reagents and antibodies

Human IL-2 Duoset ELISA kit was purchased from R&D Systems (Minneapolis, MN, USA). Mouse IgE OptEIA ELISA set was provided from BD biosciences (San Diego, CA, USA). Anti-CD69 antibodies conjugated with FITC and AnnexinV/7AAD apoptosis assay kit were purchased from BioLegend. Quanti-Max for WST assay kit and ECL for western blotting detection reagent were obtained from Biomax (Guri, Korea). Dynabeads Protein G co-immunoprecipitation (co-IP) assay kit and CellTrace^TM^ CFSE cell proliferation kit were purchased from Invitrogen by Thermo Fisher Scientific (Waltham, MA, USA). For AD induction, house dust mite (HDM) extract was provided from Greer (Lenoir, NC, USA). 2,4,-dinitrochlorobenzene (DNCB), Umi-77, phorbol 12-myristate 13-acetate (PMA) and A23187 were purchased from Sigma Chemical Co. (St. Louis, MO, USA). CNBr-Activated Sepharose 4B was obtained from GE healthcare (Chicago, IL, USA). LaboPass Q Master SYBR green was purchased from Cosmo Genetech (Seoul, Korea). Prime Script RT Master was provided from Takara (Shiga, Japan). Anti-human CD3 antibodies (Cat# BE0001-2), human CD28 antibodies (Cat# BE0248), anti-mouse CD3 antibodies (Cat# BE0001-1) and mouse CD28 antibodies (Cat# BE0015-5) for T cell stimulation were purchased from BioXcell (West Lebanon, NH, USA). Anti-bcl-2 (Cat# sc-7382) antibodies were obtained from Santa Cruz Biotechnology (Dallas, TX, USA). Anti-caspase3 (Cat# 9662), anti-caspase8 (Cat# 9746), anti-β-actin (Cat# 9746), anti-MCL-1 (Cat#94296), anti-phosphorylated MCL-1 at Ser64 (Cat#13297), anti-Bak (Cat#12105), anti-Bax (Cat#2772) antibodies were purchased from cell signaling Technology (Danvers, MA, USA).

### T cell stimulation

T cells pre-incubated with sesamin at the indicated concentration (0 to 40 µM) for 1 h at 37 °C were stimulated with immobilized anti-CD3 (10 µg/ml) antibodies and soluble anti-CD28 (2 µg/ml) antibodies for the indicated time condition (0 to 72 h) for each purpose.

### CFSE proliferation assay

Proliferative activity of T cells was measured by CFSE proliferation kit. Mouse CD4^+^ T cells pre-stained with 0.5 μM CFSE were pre-treated with 40 μM for 1 h and stimulated by anti-CD3/CD28 antibodies for 72 h. The fluorescence of CFSE was acquired by flow cytometry and mean fluorescence intensity and populations were obtained by BD flow cytometry software.

### Pull-down assay

To confirm the physical interaction between sesamin and MCL-1 as target molecule, sesamin-Sepharose 4B beads were generated as described in a previous publication 18. Briefly, activated dried powder of CNBr-Activated Sepharose 4B beads in 1 mM of HCl was added to 2 mg of sesamin in DMSO or DMSO in a coupling buffer (0.1 M NaHCO_3_ (pH 8.3) and 0.5 M NaCl). After overnight mixing, coupling buffer was discarded and replaced with 0.1 M Tris-HCl buffer (pH 8.0). This conjugate was rotated overnight, washed once with 0.1 M acetate buffer (pH 4.0) containing 0.5 M NaCl, followed by a second wash containing 0.5 M NaCl. Lysates from Jurkat T cells were incubated with control Sepharose 4B beads (bead) or sesamin-Sepharose 4B bead (SS-bead) in reaction buffer (50 mM Tris, 5 mM EDTA, 150 mM NaCl, 1 mM DTT, 0.01% NP-40, 2 mg/ml BSA, 0.02 mM PMSF and 1 μg of protease inhibitor) overnight in different ratio respectively (100:0, 50:50, 0:100). After incubation, complexes were washed with washing buffer (50 mM Tris, 5 mM EDTA, 150 mM NaCl, 1 mM DTT, 0.01% NP-40 and 0.02 mM PMSF) and eluted in SDS loading buffer. After separation into gels, MCL-1 and Bak were detected by Western blotting. The level of precipitated MCL-1 was analyzed with normalization.

### ELISA

After stimulation of T cells with anti-CD3/CD28 antibodies for 24 h in the indicated condition, supernatants were collected for enzyme-linked immunosorbent assay (ELISA). DuoSet ELISA kit was used to measure produced IL-2. For determination of immunoglobulinE (IgE) level, blood was collected from sacrificed mice and centrifuged at 7000 rpm for 5 min for obtaining serum. Serum were diluted in 1:100 before ELISA following instruction of the OptEIA ELISA set (BD biosciences, San Diego, CA, USA).

### Measurement of CD69 expression by flow cytometry

After stimulation of Jurkat T cells with anti-CD3/CD28 antibodies for 16 h in indicated conditions, cells were collected and stained with anti-CD69 antibodies conjugated with FITC for 30 min at 4 °C. Fluorescence was acquired by flow cytometry (Beckman Coulter) to detect the expression of CD69 on the surface. The mean fluorescent intensity (MFI) and histogram data were generated by using BD flow cytometry software.

### Apoptosis assay using AnnexinV and 7AAD kit

Apoptosis of Jurkat T cells was examined with a double staining method using AnnexinV conjugated with FITC and 7AAD. Briefly, incubated Jurkat T cells in indicated conditions were resuspended in 100 μl of 1X binding buffer (10 mM HEPES, 150 mM NaCl, 5 mM KCl, 5 mM MgCl_2_, 1.8 mM Cacl_2_) containing AnnexinV (20 μg/ml) and 7-AAD (1 μg/ml) and incubated at RT for 15 min. Fluorescence was acquired by flow cytometry (Beckman Coulter) and Contour plot data was generated by using BD flow cytometry software. Percentage of AnnexinV^+^7AAD^-^ (early apoptotic population) and AnnexinV^+^7AAD^+^ (late apoptotic population) was analyzed from Contour plot data. All experiments were performed in three independent biological replicates, with each sample analyzed in duplicate (technical replicates). The data shown represent the mean ± SEM from the three independent experiments.

### Mining of associated genes and predicted target proteins

To determine the target protein of sesamin in T cells, associated genes with immune-related, T cell function-related and lignan-related were mined from Genecards databanks. Using Venn diagram analysis, overlapping genes were identified in sequence. After obtaining 287 genes of lignan-related genes, 20 structure-based candidate target proteins of sesamin were predicted by Swisstargetprediction server (http://www.swisstargetprediction.ch) using canonical SMILES code (C1C2C(COC2C3=CC4=C(C=C3)OCO4)C(O1)C5=CC6=C(C=C5)OCO6) obtained from PubChem. Probabilities were computed based on a cross-validation. Among 20 candidates, only 3 candidate proteins (MCL-1, HIF1A and ALOX5) were obtained by cut off based on probabilities (> 0.1). To obtain information about interacting partners of MCL-1, we used GeneMANIA database (https://genemania.org/). The concept of biological replicates does not apply due to computational analyses using pre-existing data.

### Molecular docking analysis

Molecular docking analysis was performed using AMDock software to visualize the interaction between target proteins and small molecule and obtain binding residues and binding energy value including affinity energy, Ki value and ligand efficiency. The 3D structures used in molecular docking analysis of MCL-1, HIF1A and ALOX5 were downloaded from the PDB with IDs 5FDR, 4ZPR and 3O8Y respectively. For the binding site prediction of sesamin to the MCL-1 BH3 domain, X-ray crystal structure of human MCL-1 BH3 in complex with Bim was used (PDB ID: 2PQK). Docking poses were displayed using pyMOL software. Docking simulations were independently repeated three times using AMDock to ensure consistency of the binding poses and binding affinity scores. The most representative docking model was selected based on both binding energy and consistent pose clustering.

### Co-immunoprecipitation assay and Western blotting

Co-IP assay was performed to evaluate the interaction between MCL-1, Bax and Bak. After harvest of cells stimulated with indicated conditions, lysates were prepared by solubilizing cell pellets in RIPA buffer for 30 min at 4 °C and centrifuged. Prepared lysates were used to perform co-IP assay with co-IP Kit following the manufacture's instruction. Briefly, 20 μl of Dynabeads Protein A was added and incubated for 1 h at 4 °C to conjugate with immunoprecipitated antibodies. Conjugates were incubated with prepared lysates for ON at 4 °C and washed twice with washing buffer. Eluted samples with SDS loading buffer and heat denatured were loaded for separation on 10% SDS-PAGE gels, transferred onto nitrocellulose membranes, and blocked in 5% skim milk in TBS containing 0.1% Tween 20 (TBS-T) for 1 h. After rinse with TBS-T, membranes were incubated with indicated immunoblotted antibodies in 3% skim milk in TBS-T at 4 °C overnight. Excess primary antibodies were removed by washing the membrane four times with TBS-T. Membranes were then incubated with 0.1 μg/ml peroxidase-labeled secondary antibodies (against rabbit or mouse) for 1.5 h at RT. After four washes with TBS-T, bands were visualized with ECL western blotting detection reagents (Thermo Fisher Scientific, Waltham, MA, USA) with a Syngene G-BOX Chemi-XRQ gel documentation system. All detected bands were quantified by ImageJ and were normalized with the intensity of the loading control proteins.

### RNA Extraction and Real-Time Quantitative PCR

Total RNA was isolated from Jurkat T cells, ear tissues or cervical lymph nodes using TRIzol reagent. After quantifying the concentration of RNA, complementary DNA synthesis was conducted using 2 μg of total RNA and Prime Script RT Master. Quantitative real-time PCR using LaboPass Q Master SYBR green was performed according to manufacturer's instruction. Relative fold values were obtained using ΔΔCT method by normalization to the level of Glyceraldehyde 3-phosphate dehydrogenase (*GAPDH*) gene. The gene expression was calculated using the following equation: Gene expression = 2^-ΔΔCT^, where ΔΔCT = (CT*^Target^*-CT*^GAPDH^*). Independent experiments were performed at least three times unless otherwise indicated. The designed primer sequences were as follows:

human *IL2* (forward): CAC GTC TTG CAC TTG TCA C

(reverse): CCT TCT TGG GCA TGT AAA ACT

human *GAPDH* (forward): CGG AGT CAA CGG ATT TGG TCG TAT

(reverse): AGC CTT CTC CAT GGT GGT GAA GAC

mouse *il2* (forward): TGA GCA GGA TGG AGA ATT ACA GG

(reverse): GTC CAA GTT CAT CTT CTA GGC AC

mouse *il4* (forward): ACA GGA GAA GGG ACG CCA T

(reverse): GAA GCC GTA CAG ACG AGC TCA

mouse *il6* (forward): CCG GAG AGG AGA CTT CAC AG

(reverse): GGA AAT TGG GGT AGG AAG GA

mouse *il13* (forward): GCA ACA TCA ACA GGA CCA GA

(reverse): GTC AGG GAA TCC AGG GCT AC

mouse *il31* (forward): TCG GTC ATC ATA GCA CAT CTG GAG

(reverse): GCA CAG TCC CTT TGG AGT TAA GTC

mouse *ifng* (forward): TCA AGT GGC ATA GAT GTG GAA GAA

(reverse): TGG CTC TGC AGG ATT TTC ATG

mouse *il17* (forward): TCC CCT CTG TCA TCT GGG AAG

(reverse): CTC GAC CCT GAA AGT GAA GG

mouse *tslp* (forward): AGG CTA CCC TGA AAC TGA G

(reverse): GGA GAT TGC ATG AAG GAA TAC C

mouse *gapdh* (forward): GCA CAG TCA AGG CCG AGA AT

(reverse): GCC TTC TCC ATG GTG GTG AA

### Detection of cell viability by Trypan blue dye exclusion method and proliferation assay by WST-8 assay

Cell viability was detected with the Trypan blue dye exclusion method 26. Briefly, Jurkat T cells incubated with indicated conditions were stained with trypan blue and loaded to slides for LUNA automated cell counter (Logos Biosystems, Anyang, Korea). Based on stained cells with trypan blue, the number of live and dead cells and cell viability were automatically evaluated. For proliferation assay, Jurkat T cells incubated with indicated conditions were stained with 10 μl of WST-8 solution for 20 min. The optical density was measured at 450 nm using an iMark microplate reader (Bio-Rad Laboratories, Inc., Berkeley, CA, USA). Proliferative ratio was calculated and presented as fold increase compared to control group.

### Induction of atopic dermatitis on mouse ear

AD in BALB/c mice was induced by repeated topical application of HDM extract and DNCB on mouse ears as described previously 19-21 after randomly grouped into five mice. All *in vivo* experiments were performed using five mice per group, as approved by the IACUC. No outliers were excluded, and all animals were included in the final analysis. The sample size was selected based on ethical guidelines and commonly accepted standards for mechanistic inflammation studies, and was sufficient to detect statistically significant differences in this model. The DNCB-in duced murine model was chosen for its well-established ability to replicate the immunopathological features of human atopic dermatitis, including epidermal hyperplasia, mast cell accumulation, increased serum IgE levels, and prominent Th2/Th17-skewed immune responses. While this model primarily represents the acute exacerbation phase, it remains highly relevant for evaluating immune modulation strategies that target effector T cells. After mice were anesthetized by injection of avertin to induce AD, surfaces of both ear lobes were stripped four times with a surgical tape (Seo-il chemistry, Hwasung, Korea) and were painted with 10 μl of DNCB (1%) for initial sensitization on Day -7. After seven days later, ears were painted with 10 μl of DNCB (1%) on Day 0. Three days later, ears were painted with 10 μl of HDM extract (10 mg/ml) on Day 3. The HDM extract/DNCB treatment was repeated weekly for 4 weeks. Sesamin or tofacitinib was administered orally by gavage five times per week (Monday through Friday) for four consecutive weeks, starting from day 0 after the initial DNCB sensitization, resulting in a total of 20 doses. Ear thickness was measured after application of HDM extract or DNCB by using a dial thickness gauge (Kori Seiki MFG Co., Tokyo, Japan). Body weight was assessed at day 0, 14 and 28 post-induction and scratching number was evaluated at day 28 post-induction. Animals were sacrificed at day 28.

### Evaluation of clinical score

Degree of inflammation and dermal papilla on the H&E-stained slide was assessed along with following standards. The standard of clinical score for inflammation is as below; 0: no signs of inflammation are observed. 1: mild inflammatory response, a small number of immune cells infiltration and minor changes in the tissue are observed. 2: Clear inflammatory response, a moderate level of immune cell infiltration and moderate changes in the tissue are observed. Early fibrosis resulted from hypertrophy of the epidermis are also detected. 3: Severe inflammatory response, a large number of immune cells infiltration with serious tissue changes and severe hypertrophy of the epidermis observed. The standard of clinical score for dermal papilla is as below; 0: no changes in dermal papillae are observed. 1: mild changes including expanded or elongated dermal papillae and mild cellular infiltration are observed. 2: Clear expansion and elongation of dermal papillae and structural changes in the dermis around the papillae are observed. 3: Severe expansion and elongation of dermal papillae and a large amount of inflammatory cellular infiltration along with serious structure changes are observed. For measuring scratching event, each mouse was individually observed in a quiet environment, and the number of scratching episodes was manually counted for a 1-minute period using a stopwatch on Day 28. Scratching was defined as repeated hind limb movements directed toward the affected ear skin. The assessment was performed by an observer blinded to the experimental groups to minimize bias. All mice were evaluated individually, and the scratching counts were analyzed.

### Histopathological analysis

After sacrifice, ear tissues were collected for histopathological analysis. Removed ear tissues were fixed in 10% paraformaldehyde and embedded in paraffin. Paraffin-embedded tissues were sliced into 5-μm-thick sections, deparaffinized, and stained with hematoxylin and eosin (H&E). H&E-stained slides were used to measure the thickness of dermis and epidermis and clinical score of inflammation and dermal papilla as previous publications 22-25. To determine the number of infiltrating mast cells, sliced sections were stained with 0.01% toluidine blue and infiltrated mast cells at randomly selected sites were counted. Histological scoring of inflammation and dermal papilla structure was performed by two independent, board-certified pathologists in a blinded manner. The evaluators used predefined scoring criteria, and average values were used for analysis.

### Statistics

Mean values were calculated from the data obtained from three separate *in vitro* experiments performed on separate days. For *in vivo* study, mean values were obtained from five each mouse and indicated individual results as hollow circle. For experiments involving multiple group comparisons, such as cytokine mRNA expression or serum IgE quantification, we applied one-way ANOVA followed by Tukey's post hoc test to adjust for multiple comparisons. For experiments involving two groups, we used unpaired two-tailed Student's t-tests. * indicates if differences between groups were considered significant (*P* < 0.05).

## Results

### Sesamin suppresses T cell activation marker and cytokine production

Since sesamin is known to modulate inflammation in other immune cells, we first examined *IL-2* mRNA levels in the presence of sesamin to determine whether it can regulate T cell function. Sesamin strongly and dose-dependently downregulated the mRNA levels of *IL-2*, a marker of T cell activation stimulated by immobilized anti-CD3 and anti-CD28 antibodies, in human T cells (left) and mouse CD4^+^ T cells (right) (Fig. 1B). We examined the suppressive effect of sesamin on IL-2 production in T cells stimulated by PMA/A23187 (Supplementary Fig. 1A); this revealed that 40 µM sesamin was the optimal concentration to suppress IL-2 production. Sesamin dose-dependently reduced IL-2 production by activated human T cells (left) and mouse CD4^+^ T cells (right) after TCR ligation, based on ELISA (Fig. 1C). Pretreatment with 40 μM sesamin time-dependently reduced IL-2 production by activated T cells (Supplementary Fig. 1B). Considering that CD69 on the surface of T cells is the earliest marker of activated T cells 26, we also tested whether pretreatment with sesamin controls CD69 expression on the surface of activated T cells. Pretreatment of activated T cells with 40 μM sesamin reduced CD69 expression (Fig. 1D). This supports the hypothesis that sesamin may act as a natural inhibitor of T cell-mediated inflammation by interfering with early activation signals. Proliferative activity was examined to determine whether sesamin inhibits T cell proliferation. Pretreatment with 40 μM sesamin reduced CFSE fluorescence intensity in proliferating T cells, inhibiting activated T cell division. These results suggest that sesamin inhibits activated T cell function, IL-2 production, CD69 expression, and proliferation in humans and mice.

### Sesamin interacts physically with MCL-1, a predicted target molecule in T cells

**Figure 2:**
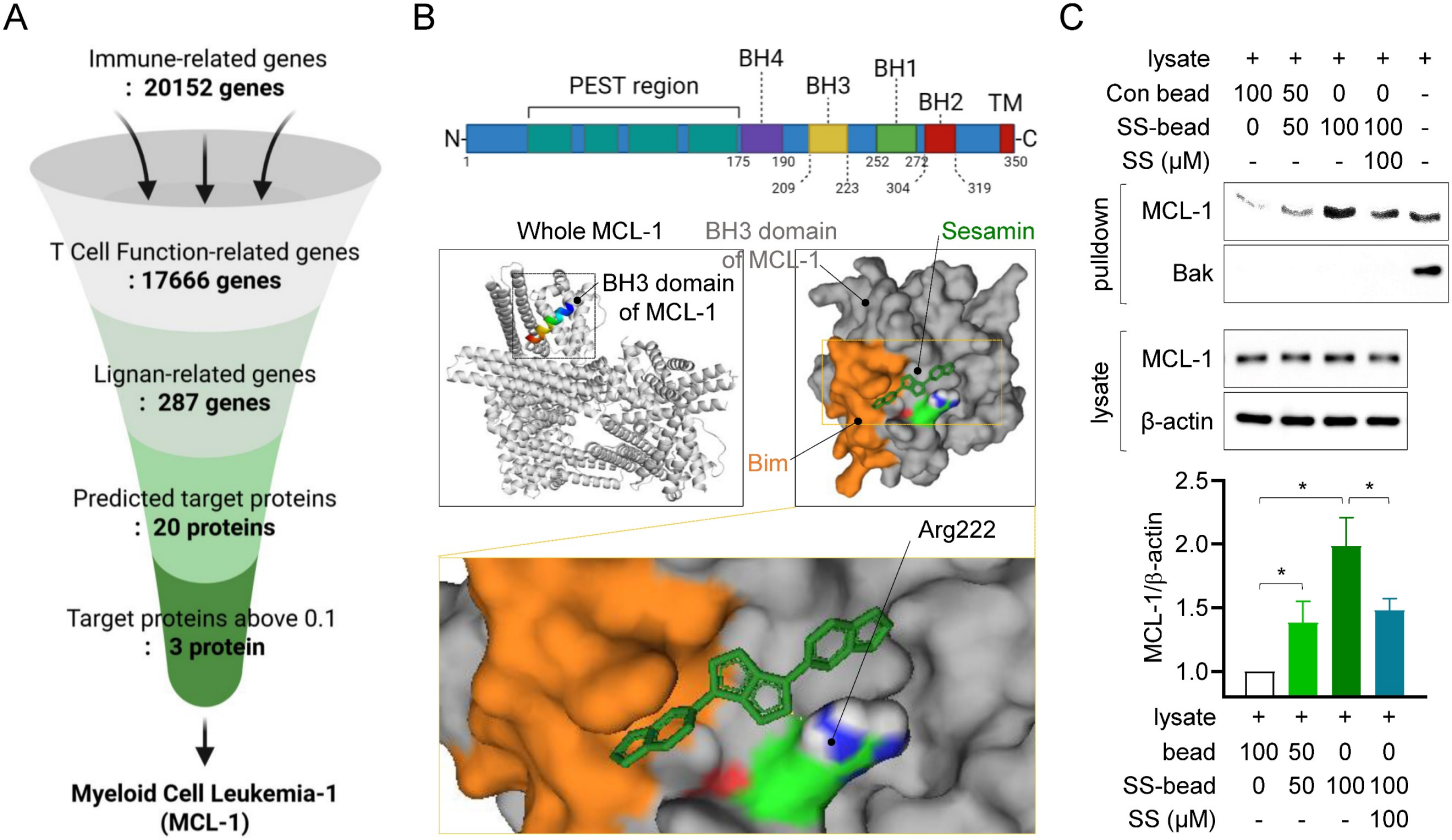
** Sesamin interacts physically with MCL-1, a predicted target molecule in T cells.** (A) The flow chart how target proteins of sesamin were discovered from Genecards databank and swisstargetprediction analysis. (B) The structure of MCL-1 active domains (above) and molecular docking between BH3 domain of MCL-1 and sesamin by AMdock and pyMOL software. BH3-only protein Bim is marked as orange color following as sequence. A binding residue of sesamin at Arg 222 is marked as blue color. (C) Resting Jurkat T cells (1 X 10^6^) were lysed with RIPA buffer for pull-down assay. Lysates were incubated with control bead (Con bead) or/and bead conjugated with sesamin (SS-bead) and precipitated by centrifugation. Con beads and SS-beads were mixed in 100:0, 50:50, 0:100 ratios, respectively, to assess the dose-dependent enrichment of MCL-1 and Bak. Mcl-1 and Bak were detected from precipitated lysate by bead centrifugation (pull-down) and MCL-1 and β-actin were detected from whole lysate. The expression of detected MCl-1 from precipitated lysate by bead centrifugation (pull-down) was normalized with the expression of β-actin from whole lysate. Bar graph was presented as fold increase. All results are expressed as mean ± SEM of three independent experiments. Statistical comparisons among groups were performed using one-way ANOVA with Tukey's post hoc test. A p-value less than 0.05 was considered statistically significant (*, P < 0.05).

To determine the mechanisms underlying the inhibition of T cell function by sesamin, we first screened for genes associated with immune responses in the GeneCards databank (Fig. 2A). This *in silico* analysis identified 20152 immune-related genes, of which 17666 participate in T cell function. Among these 17666 genes, 287 genes were identified as lignan-related. Among these 287 genes, 20 target proteins were identified as predicted binding partners of sesamin, based on their protein structure, using the SwissTargetPrediction database. Among these 20 proteins, three had a probability value above the 0.1 cutoff (Supplementary Fig. 2A). Of these, Myeloid Cell Leukemia-1, an anti-apoptotic BCL-2 family protein (MCL-1) exhibited the highest affinity energy (10.2 kcal/mol), lowest Ki value (33.37 nM) and highest ligand efficiency (0.39), based on molecular docking analysis, and was selected as a target of sesamin in T cells (Supplementary Fig. 2B, 2C). Docking analysis demonstrated sesamin interacts with a residue at Arg 222 in BH3 domain of MCL-1 where recognized to play a critical role for heterodimerization with pro-apoptotic BH3-only proteins (Fig. 2B). To confirm whether sesamin binds physically to MCL-1 in T cells, an *in vitro* experiment with pull-down assays was performed using the CNBr-activated Sepharose 4B bead system. Notably, this experiment was conducted using resting Jurkat T cells, as MCL-1 is constitutively expressed even in the absence of activation. This allowed us to test whether sesamin could engage MCL-1 prior to T cell stimulation, thereby providing a mechanistic basis for the enhanced sensitivity to apoptosis. Moreover, this approach was consistent with earlier functional assays in which sesamin was pretreated in resting cells before activation (Fig 1), reinforcing the physiological relevance of our experimental model. MCL-1 was dose-dependently precipitated with the 2 mg of sesamin-conjugated Sepharose 4B beads but not with the control Sepharose 4B beads (Fig. 2C). When Sepharose 4B beads were mixed with the T cell lysates in the presence of soluble sesamin (100 μM) as a competitive inhibitor, MCL-1 precipitation was significantly reduced. Bak, a partner protein that heterodimerizes with MCL-1, was not detected via the pull-down assay using sesamin-conjugated Sepharose 4B beads. These results strongly indicate that sesamin physically interacts with the active site of MCL-1 in T cells.

### Sesamin inhibits MCL-1 activity in activated T cells, controlling T cell activation

**Figure 3:**
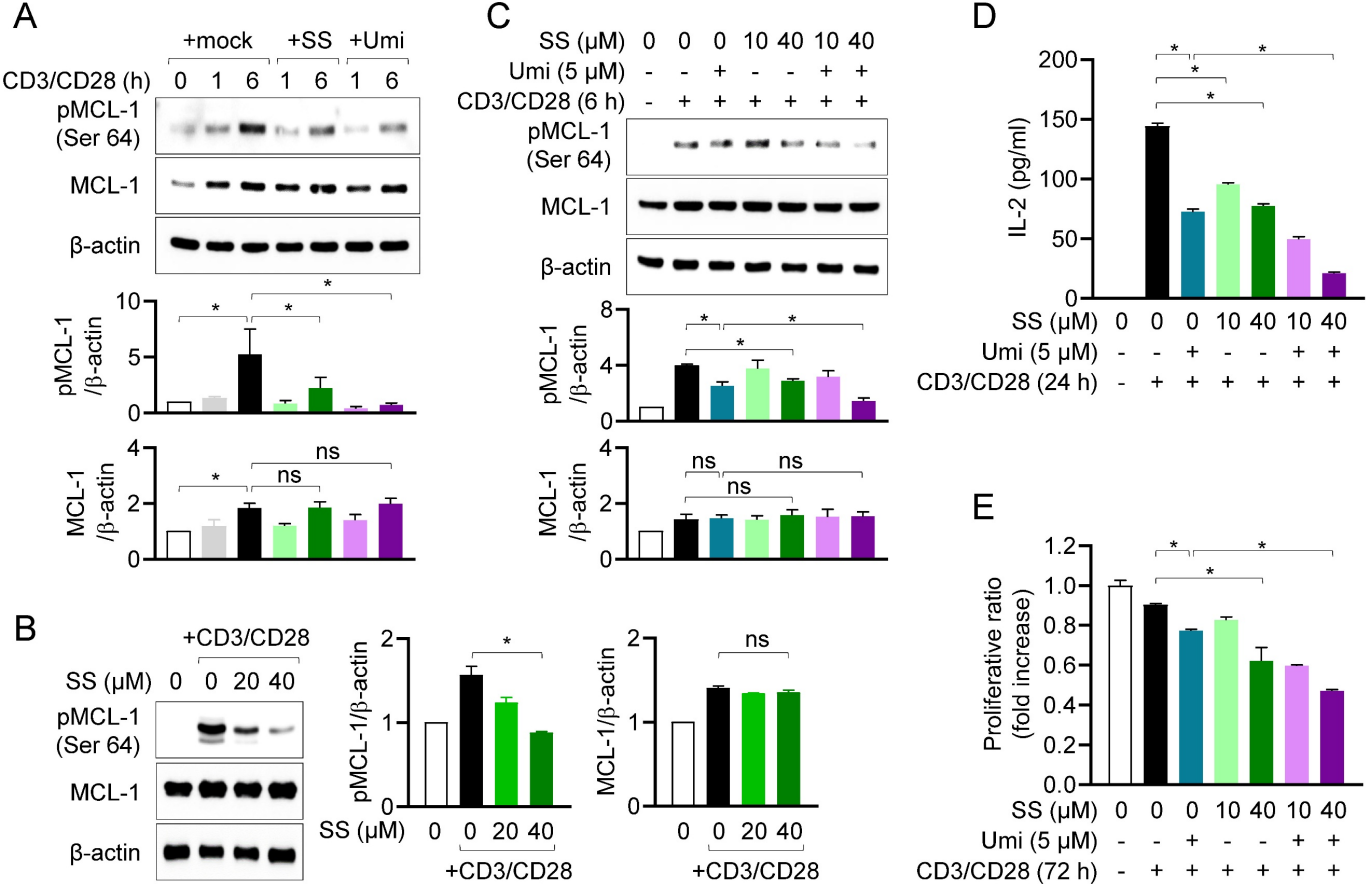
** Sesamin inhibits MCL-1 activity in activated T cells, controlling T cell activation.** (A) Jurkat T cells (1 X 10^6^/well, 12-well plate) pre-treated with 40 μM sesamin or 5 μM Umi-77 for 1 h were stimulated with immobilized anti-CD3 (10 μg/ml) and soluble anti-CD28 (2 μg/ml) antibodies for 1 to 6 h. Harvested cells were lysed in RIPA buffer and expressions of phosphorylated MCL-1 at Ser64 and MCL-1 were detected by Western blotting analysis. Expressions were normalized with the expression of β-actin. (B) Mouse CD4^+^ T cells (3 X 10^6^/well, 12-well plate) pretreated with 20 or 40 μM sesamin for 1 h were stimulated with anti-CD3 (10 μg/ml) and soluble anti-CD28 (2 μg/ml) antibodies for 6 h. Harvested cells were lysed in RIPA buffer and expressions of phosphorylated MCL-1 at Ser64 and MCL-1 were detected by Western blotting analysis. Expressions were normalized with the expression of β-actin. (C-E) Jurkat T cells (1 X 10^6^/well, 12-well plate) pre-treated with 10 or 40 μM sesamin for 1 h and then 5 μM Umi-77 for additional 1 h were stimulated with immobilized anti-CD3 (10 μg/ml) and soluble anti-CD28 (2 μg/ml) antibodies for 6 h (C), 24 h (D) and 72 h (E). Harvested cells were lysed in RIPA buffer and the expression of phosphorylated MCL-1 at Ser64 and MCL-1 were detected by Western blotting analysis. Expressions were normalized with the expression of β-actin (C). The amount of released IL-2 was evaluated by ELISA from harvested supernatants (D). WST-8 assay was performed to obtain proliferative ratio compared to control. OD value of control group was considered as 1X and fold increase was presented compared to control group (E). All results are expressed as mean ± SEM of three independent experiments. Statistical comparisons among groups were performed using one-way ANOVA with Tukey's post hoc test. A p-value less than 0.05 was considered statistically significant (*, P < 0.05).

We next examined whether the interaction between sesamin and MCL-1 affects MCL-1 activity in T cells. Using western blotting, we measured MCL-1 phosphorylation at site Ser64, a marker of MCL-1 activity 27, in the presence of sesamin. In human T cells, MCL-1 expression was enhanced by TCR-mediated stimulation, and MCL-1 was fully phosphorylated within 6 h (Fig. 3A). Pretreatment with 40 μM sesamin markedly suppressed MCL-1 phosphorylation at Ser64, in a similar manner to the suppression observed after pretreatment with Umi-77 in human T cells. The inhibitory effects of sesamin on MCL-1 phosphorylation were confirmed in mouse CD4^+^ T cells, in which pretreatment with 20 and 40 µM sesamin significantly downregulated MCL-1 phosphorylation in dose-dependent manner, but not its expression (Fig. 3B). To validate the regulatory effect of sesamin on MCL-1 activity in T cells, a co-treatment assay was performed using Umi-77. Pretreatment of T cells with Umi-77 and 20 or 40 μM sesamin dose-dependently suppressed MCL-1 activity more than treatment with Umi-77 alone (Fig. 3C). Next, we examined whether sesamin and Umi-77 treatment reduce MCL-1 activity, thus downregulating T cell function. ELISA revealed that treatment with Umi-77 and sesamin reduced more IL-2 production in dose-dependent manner by activated T cells compared to treatment with Umi-77 alone (Fig. 3D). Pretreatment with sesamin inhibited proliferation of activated T cells in dose-dependent manner but Umi-77 and 40 μM sesamin had a stronger effect on the proliferation of activated T cells than pretreatment with Umi-77 alone (Fig. 3E). These results suggest that, in humans and mice, sesamin inhibits MCL-1 activity, thereby inhibiting T cell activation.

### Sesamin disrupts the interaction of MCL-1 with Bak in activated T cells

**Figure 4:**
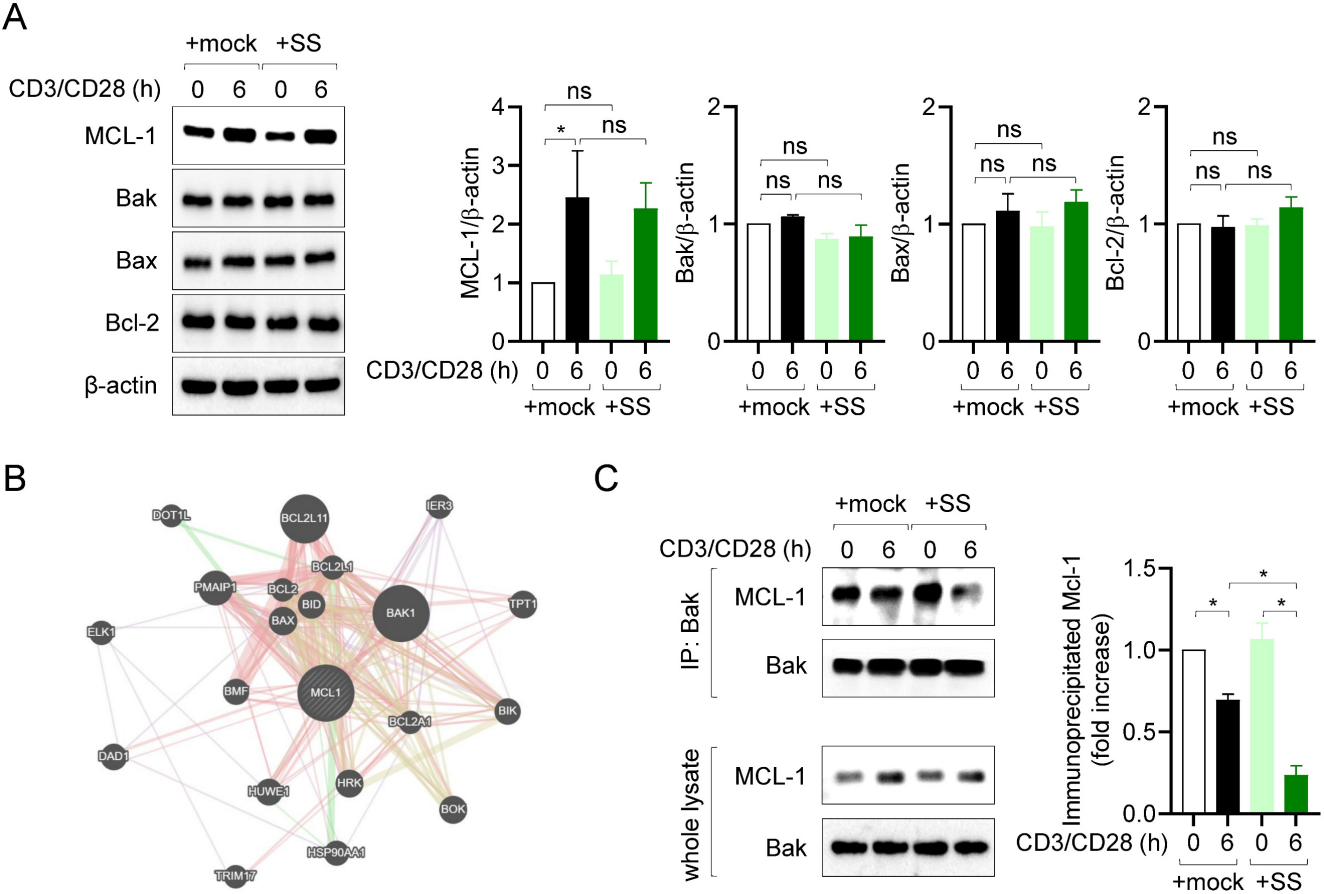
** Sesamin disrupts the interaction of MCL-1 with Bak in activated T cells.** (A) Jurkat T cells (5 X 10^5^/well, 12-well plate) pre-treated with 40 μM sesamin for 1 h were stimulated with immobilized anti-CD3 (10 μg/ml) and soluble anti-CD28 (2 μg/ml) antibodies for 6 h. Harvested cells were lysed in RIPA buffer and the expression of MCL-1, Bak, Bax and Bcl-2 was detected by Western blotting analysis. Expressions were normalized with the expression of β-actin. (B) A prediction result showing binding partners of MCL-1 by GeneMANIA database. (C) Co-IP assay experiments were performed with lysates demonstrated in (A). IP experiments with anti-Bak antibodies were performed and the expression of precipitated MCL-1 and Bak was detected by Western blotting. Precipitated MCL-1 was normalized with the expression of precipitated Bak and the expression of MCL-1 in whole lysate. All results are expressed as mean ± SEM of three independent experiments. Statistical comparisons among groups were performed using one-way ANOVA with Tukey's post hoc test. A p-value less than 0.05 was considered statistically significant (*, P < 0.05).

MCL-1 plays a pivotal role in regulating the apoptotic pathway, by forming heterodimers with proapoptotic Bcl-2 family proteins in several cell types 28. Therefore, we first examined the expression of MCL-1, Bak, Bax, and Bcl-2 in resting and activated T cells. Consistent with prior findings 29,30, MCL-1 expression was upregulated, whereas that of Bak, Bax, and Bcl-2 was not affected, by TCR stimulation or pretreatment with sesamin (Fig. 4A). Next, we evaluated which proapoptotic Bcl-2 family protein is mainly interacted with MCL-1 in T cells. A result from GeneMANIA database predicted Bak as a main partner of MCL-1 (Fig. 4B). To investigate whether T cell stimulation and pretreatment with 40 μM sesamin influence the interaction between MCL-1 and Bak, co-immunoprecipitation (co-IP) experiments were performed. T cell stimulation alone partially reduced the interaction between MCL-1 and Bak, as observed by co-IP. However, pretreatment with 40 μM sesamin resulted in a markedly greater decrease in this interaction (Fig. 4C), suggesting that sesamin facilitates the dissociation of the MCL-1-Bak complex and thereby promotes apoptosis in activated T cells. These results suggest that inhibiting MCL-1 activity via sesamin disrupts or weakens MCL-1-Bak interaction, thereby facilitating apoptosis in activated T cells.

### Sesamin selectively induces caspase-dependent apoptosis in activated T cells

**Figure 5:**
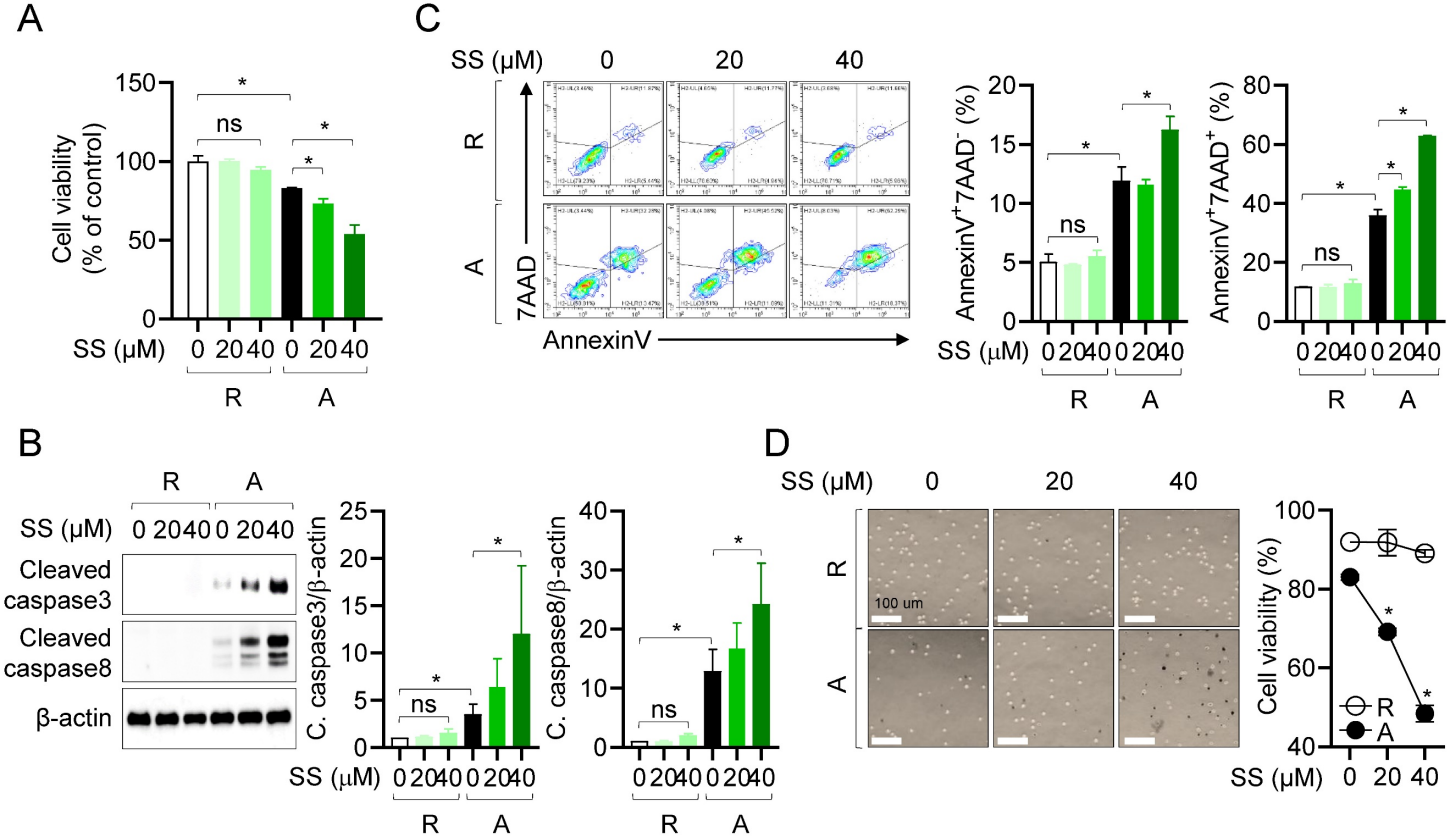
** Sesamin selectively induces caspase-dependent apoptosis in activated T cells.** (A) Jurkat T cells (1 X 10^4^/well, 96-well plate) pre-treated with 40 μM sesamin for 1 h were stimulated with immobilized anti-CD3 (10 μg/ml) and soluble anti-CD28 (2 μg/ml) antibodies for 72 h. WST-8 assay was performed to obtain cell viability. (B) Jurkat T cells (1 X 10^6^/well, 12-well plate) pre-treated with 20 or 40 μM sesamin for 1 h were stimulated with immobilized anti-CD3 (10 μg/ml) and soluble anti-CD28 (2 μg/ml) antibodies for 48 h. Harvested cells were lysed in RIPA buffer and the expression of cleaved caspase3 and caspase8 were detected by Western blotting analysis. Expressions were normalized with the expression of β-actin. (C) Jurkat T cells (5 X 10^5^/well, 12-well plate) pre-treated with 20 or 40 μM sesamin for 1 h were stimulated with immobilized anti-CD3 (10 μg/ml) and soluble anti-CD28 (2 μg/ml) antibodies for 48 h. Harvested cells were stained with AnnexinV and 7AAD for AnnexinV apoptosis assay. From Contour Plot, the population of AnnexinV^+^7AAD^-^ and AnnexinV^+^7AAD^+^ was analyzed. (D) The DIC images of Jurkat T cells from Trypan blue exclusion method. Black dots indicate Trypan blue-stained cells in the images. White bar represents 100 μm. All results are expressed as mean ± SEM of three independent experiments. Statistical comparisons among groups were performed using one-way ANOVA with Tukey's post hoc test. A p-value less than 0.05 was considered statistically significant (*, P < 0.05).

We confirmed that treatment with 40 μM sesamin before and during stimulation with anti-CD3/CD28 antibodies reduced heterodimerization between MCL-1 and pro-apoptotic protein Bak in activated T cells (Fig. 4). In T cells, sesamin binds to the active site of MCL-1 and inhibits its activity, reducing its dimerization with pro-apoptotic proteins, and thus potentially accelerating the apoptotic pathway. To determine whether the suppressive effects of sesamin on T cell activation were due to specific immune modulation or general cytotoxicity (Figure 1), we assessed cell viability and apoptosis using AnnexinV/PI staining. The WST-8 assay revealed that sesamin dose-dependently and substantially reduced the viability of activated but not resting T cells (Fig. 5A). To assess whether sesamin kills activated T cells by inducing apoptosis, we measured the expression of cleaved caspase 3 and 8. Western blotting revealed that, in T cells, cleavage of caspase 3 and 8 was substantially elevated 48 h after treatment with 20 or 40 μM sesamin before and during stimulation with anti-CD3/CD28 antibodies (Fig. 5B). In activated human T and mouse CD4^+^ T cells, cleavage of caspase 3 and 8 was time-dependently enhanced by treatment with 40 μM sesamin before and during stimulation with anti-CD3/CD28 antibodies (Supplementary Fig. 3A, 3B). Annexin V/7-AAD assays confirmed that apoptosis was induced 48 h after TCR-mediated stimulation, and treatment with 20 or 40 μM sesamin before and during stimulation with anti-CD3/CD28 antibodies enhanced apoptosis in both the early (AnnexinV^+^7AAD^-^) and late (AnnexinV^+^7AAD^+^) phases (Fig. 5C). In resting T cells, treatment with 40 μM sesamin before and during stimulation with anti-CD3/CD28 antibodies did not affect apoptosis. A time-dependent experiment confirmed that sesamin upregulated apoptosis in activated but not in resting T cells (Supplementary Fig. 4). A trypan blue exclusion assay was performed to assess T cell viability during AICD. Cell death was enhanced in activated T cells 72 h after treatment with 40 μM sesamin before and during stimulation with anti-CD3/CD28 antibodies (Fig. 5D). Blue-stained activated T cells were more abundant following treatment with sesamin before and during stimulation with anti-CD3/CD28 antibodies (Fig. 5D). These results suggest that, in humans and mice, sesamin induces apoptosis in activated, but not resting, T cells.

### Orally administered sesamin ameliorates AD-model symptoms *in vivo*

**Figure 6:**
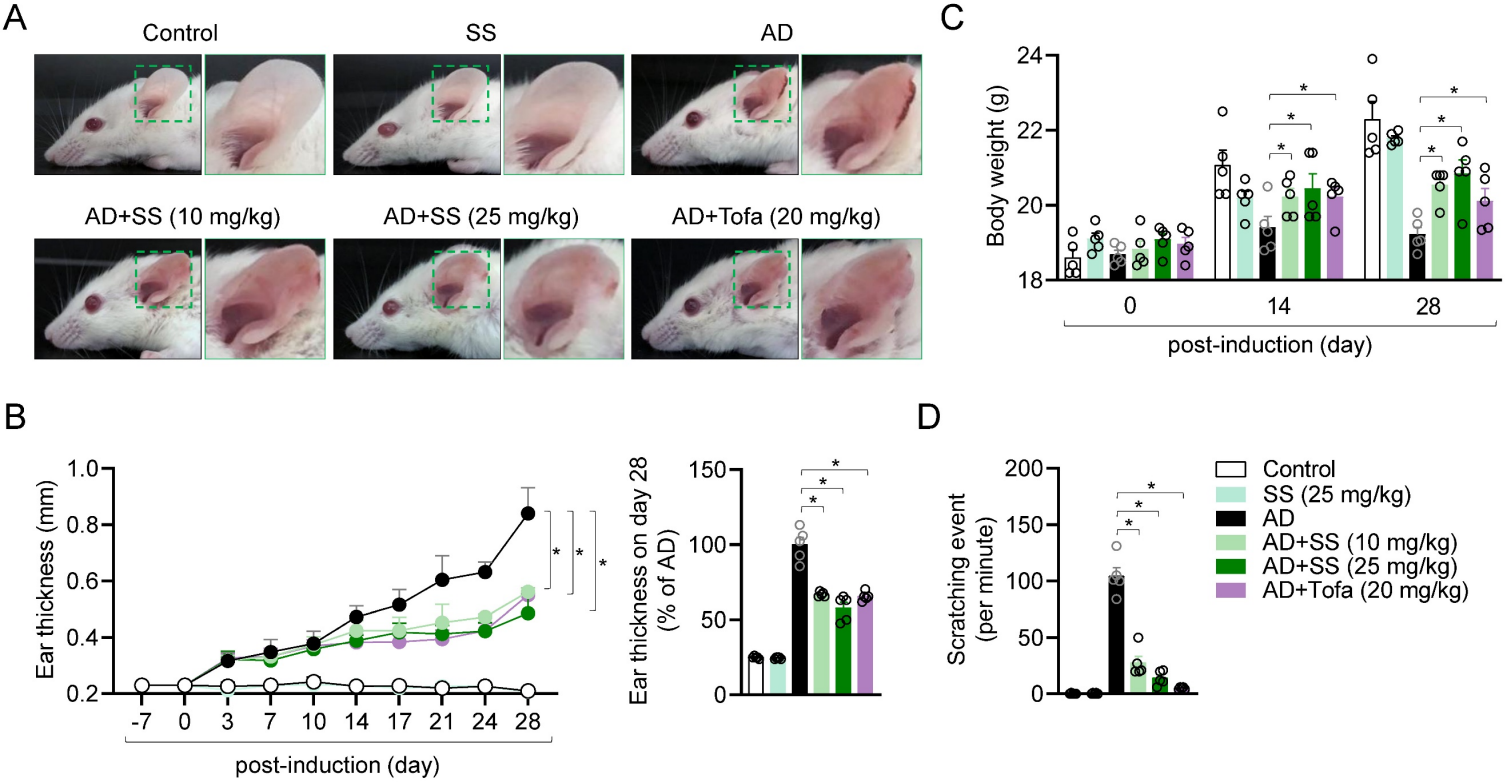
** Orally administered sesamin ameliorates AD-model symptoms *in vivo.*** (A) Representative images of mouse ears at day 28 post-induction. (B) Changes of ear thickness of each mouse group during induction (left) and ratio of ear thickness at day 28 (right). (C) Body weight of each mouse at day 0, 14 and 28 post-induction. (D) Scratching number of each mouse per 1 min. Control: Untreated mice reveiving only vehicle (0.5% CMC) without AD induction or sesamin administration. SS: Mice administrated sesamin (25 mg/kg) without AD induction to assess the baseline immunological effect of sesamin. AD: Mice sensitized and challenged with DNCB/HDM to induce AD, receiving vehicle only. AD+SS (10 mg/kg): Mice treated with DNCB/HDM and administered sesamin at 10 mg/kg to evaluate low-dose therapeutic effects. AD+SS (25 mg/kg): Mice treated with DNCB/HDM and administered sesamin at 25 mg/kg to evaluate high-dose therapeutic effects. AD+Tofa (20 mg/kg): Mice treated with DNCB/HDM and administered tofacitinib at 20 mg/kg as a positive control for anti-inflammatory efficacy. All results are expressed as mean ± SEM of three independent experiments. Statistical comparisons among groups were performed using one-way ANOVA with Tukey's post hoc test. A p-value less than 0.05 was considered statistically significant (*, P < 0.05).

To determine whether the mechanism identified *in vitro* applies in T cell-driven diseases *in vivo*, an AD model was generated. Treatment of ear tissue with HDM extract and DNCB treatment has been reported to induce systemic manifestations of AD (18,20-23,31). Ear tissue condition was monitored for 28 d after induction of AD. Ear tissue from the AD group exhibited the general symptoms of AD, including redness, swelling, crusting, and lichenification (Fig. 6A). Treatment with 25 mg/kg sesamin significantly reduced ear thickness in AD mice by approximately 57%, compared to a 62% reduction observed in the tofacitinib-treated group (Fig. 6B). To elucidate physiological improvements in the AD model, we evaluated changes in body weight and the number of ear scratches per minute on day 28 post-induction. Orally administered sesamin markedly reversed the reduction in body weight in the AD group (Fig. 6C) and reduced ear scratching (Fig. 6D). These results suggest that the orally administered sesamin improves AD *in vivo*.

### Orally administered sesamin attenuates pathological manifestations in the AD model

**Figure 7:**
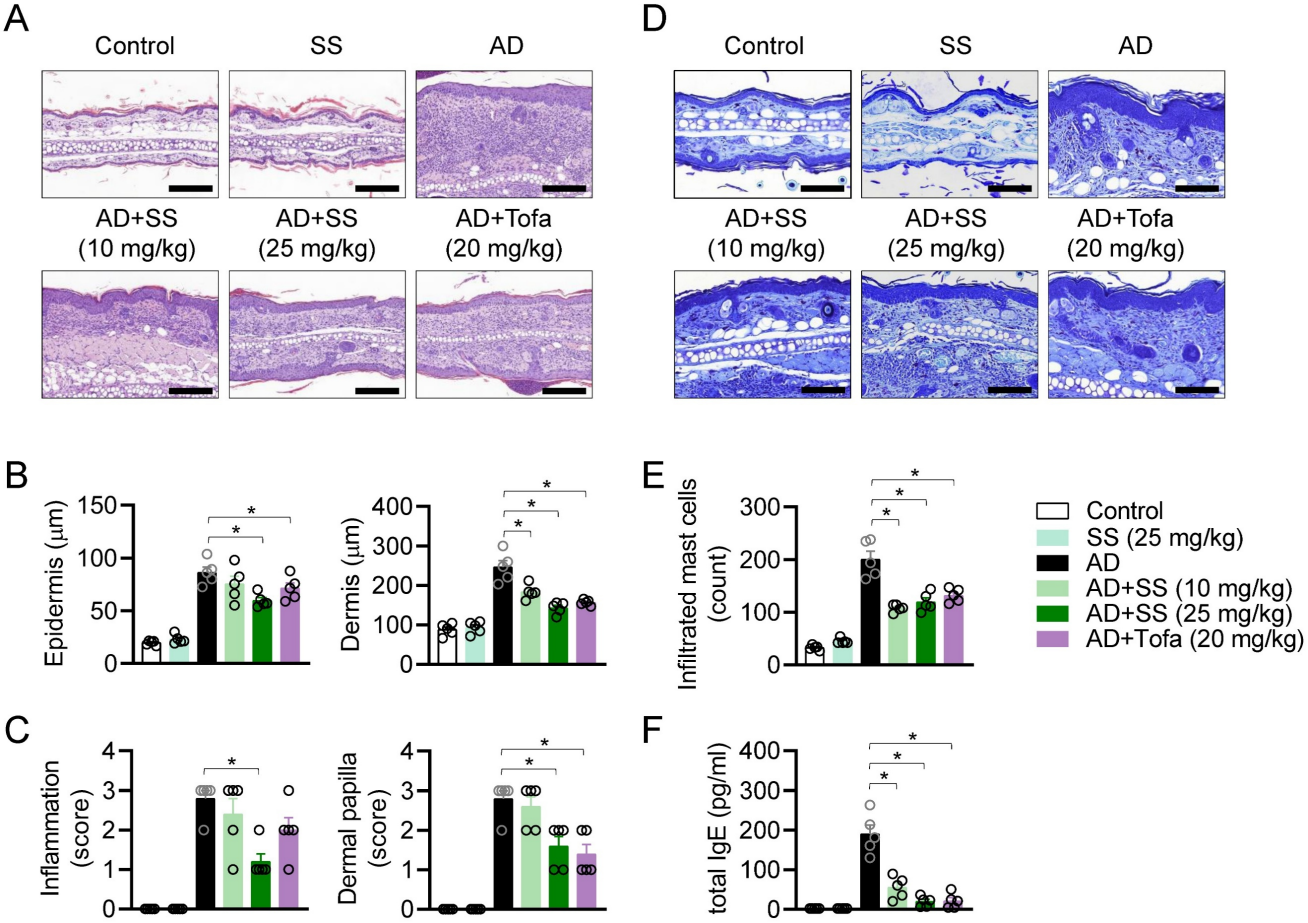
** Orally administered sesamin attenuates pathological manifestations in the AD model.** (A) Representative H&E histopathological images of mice ears. Black bar represents 200 μm. (B) Thickness of epidermis and dermis of each mouse from (A). (C) Clinical scores according to inflammation and dermal papilla of each mouse were evaluated from (A). Measuring standard is described in Materials and methods. (D) Representative Toluidine blue histopathological images of mice ears. Black bar represents 400 μm. (E) The number of infiltrated mast cells to the lesion was counted. (F) Levels of total IgE in serum were assessed by ELISA. All results are expressed as mean ± SEM of three independent experiments. Statistical comparisons among groups were performed using one-way ANOVA with Tukey's post hoc test. A p-value less than 0.05 was considered statistically significant (*, P < 0.05).

Histopathological analysis was performed to assess the effects of orally administered sesamin on AD *in vivo*. Ear tissue was stained with H&E to analyze changes in skin structure, cellularity, and inflammatory condition (Fig. 7A). Epidermal and dermal thickness were remarkably reduced by orally administered sesamin relative to that in the untreated AD group (Fig. 7B). Inflammation and the formation of dermal papillae, typical structural changes observed in AD, were attenuated by orally administered sesamin (Fig. 7C). In the AD model, the ear tissue stained blue following toluidine blue staining, because infiltrating granule-positive mast cells play a critical role in the pathogenesis of AD (Fig. 7D). Orally administered sesamin reduced the infiltrating mast cell count in the lesion relative to the that in untreated AD group (Fig. 7E), and reduced serum IgE, which activates degranulation of mast cells during the allergic response and is a hallmark of AD (Fig. 7F). These results suggest that orally administered sesamin attenuates the pathological manifestations of AD, including structural changes and mast cell infiltration, *in vivo*.

### Orally administered sesamin reduces ear-tissue atopic gene expression in the AD model

**Figure 8:**
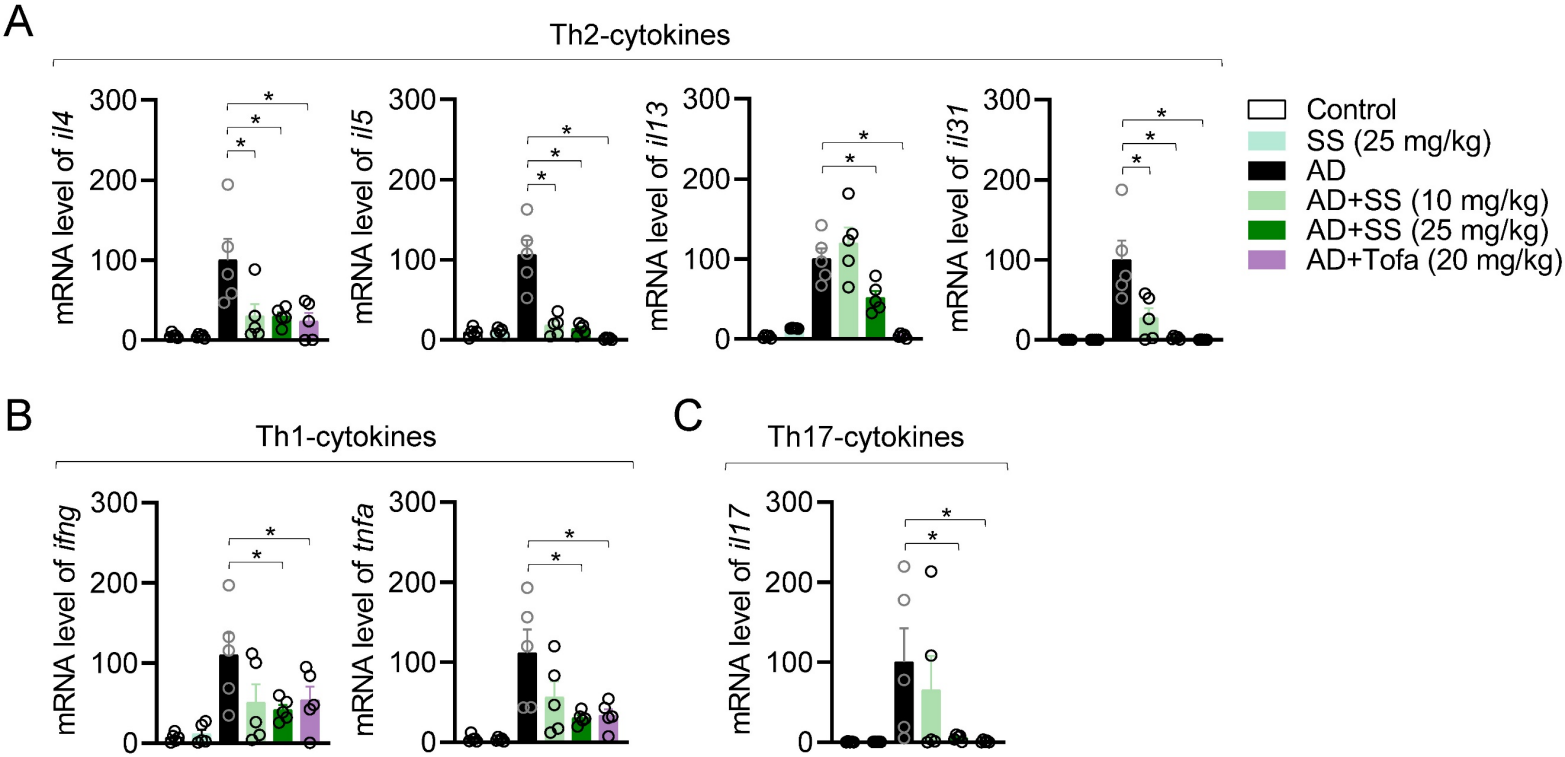
** Orally administered sesamin reduces ear-tissue atopic gene expression in the AD model.** (A-C) mRNA levels of Th2 type cytokines (A), Th1 type cytokines (B) and Th17 type cytokines (C) from ear tissues of each mouse. The expression of target genes was normalized with the expression of *Gapdh*. All results are expressed as mean ± SEM of three independent experiments. Statistical comparisons among groups were performed using one-way ANOVA with Tukey's post hoc test. A p-value less than 0.05 was considered statistically significant (*, P < 0.05).

To elucidate whether orally administered sesamin reduced atopic gene expression, mRNA levels in ear tissue were analyzed via qPCR. This revealed that orally administered sesamin reduced the mRNA levels of Th2 cytokines including *il4*, *il5*, *il13*, and *il31* relative to those in the AD group (Fig. 8A), reduced those of Th1 cytokines (*ifng* and *tnfa*) and Th17 cytokines (*il17*) (Fig. 8B, 8C), but did not alter those of *il6* and the keratinocyte chemokine *tslp* (Supplementary Fig. 5A, 5B). These results suggest that orally administered sesamin reduces atopic gene expression in AD-affected ear tissue *in vivo*.

### Orally administered sesamin ameliorates the systemic immune response associated with T cell activation in the AD model

**Figure 9:**
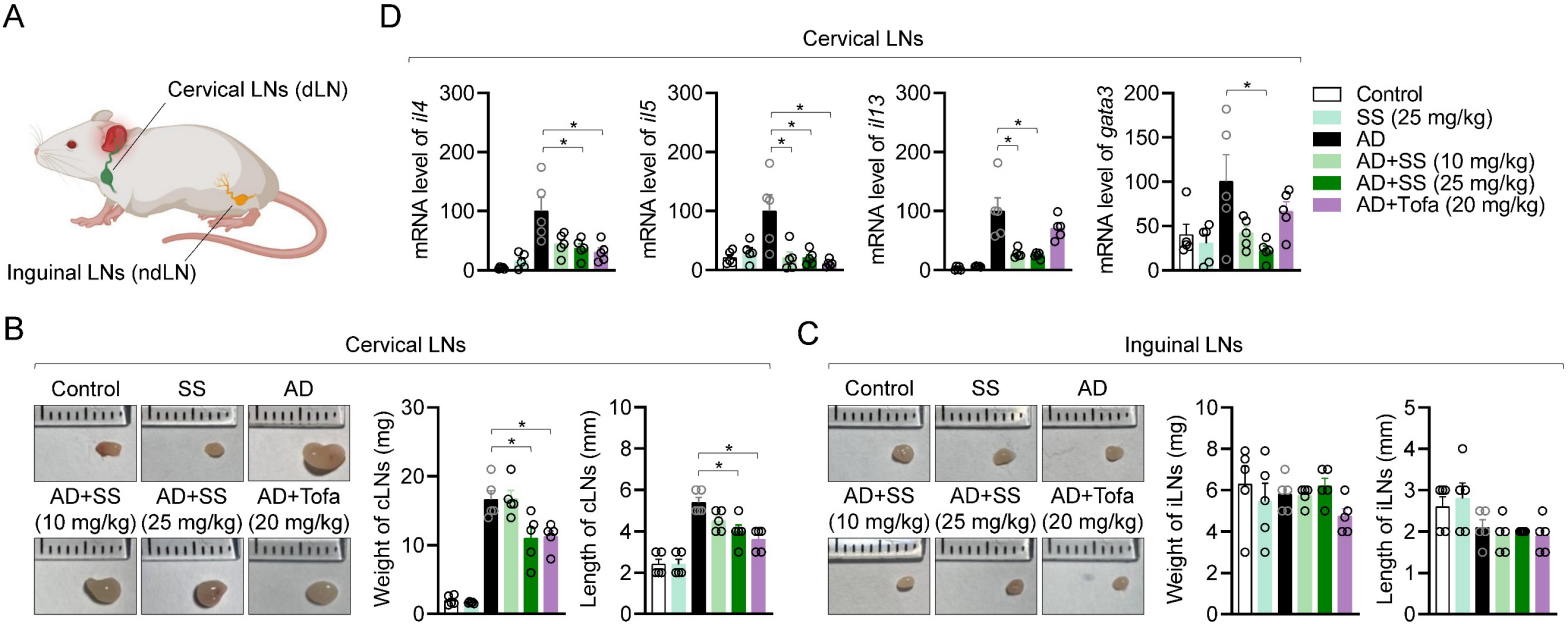
** Orally administered sesamin ameliorates the systemic immune response associated with T cell activation in the AD model.** (A) Anatomical localization of cervical lymph nodes and inguinal lymph nodes on mouse. (B) Representative images of cervical lymph nodes of each mouse group (left), their weights (middle) and lengths (right). (C) Representative images of inguinal lymph nodes of each mouse group (left), their weights (middle) and lengths (right). (D) mRNA levels of Th2 type cytokines (IL-4, IL-5 and IL-13) and master transcription factor of Th2 effector cells (GATA3) from cervical lymph nodes of each mouse. The expression of target genes was normalized with the expression of *Gapdh*. All results are expressed as mean ± SEM of three independent experiments. Statistical comparisons among groups were performed using one-way ANOVA with Tukey's post hoc test. A p-value less than 0.05 was considered statistically significant (*, P < 0.05).

Several publications have revealed that systemic inflammatory responses associated with T cell activation occur in cervical lymph nodes (cLNs) than inguinal lymph nodes (iLNs) due to close distance during atopic dermatitis at ear tissue (Fig. 9A). To determine whether orally administered sesamin systemically affects T-cell immunity during AD development, we compared the weights and lengths of cLNs and iLNs. Representative images of swollen and enlarged cLNs from the AD group are shown in Fig. 9B. *In vivo*, orally administered sesamin reduced cLN weight and length (Fig. 9B), without significantly altering iLN morphology or weight or length (Fig. 9C). To clarify whether it leads to improvement of peripheral T cell-mediated immune responses in AD, atopic gene mRNA levels were analyzed in cLN tissue. Orally administered sesamin reduced mRNA levels of Th2 cytokines, including *il4*, *il5*, and *il13*, and the Th2 master transcription factor *gata3* (Fig. 9D). These results suggest that orally administered sesamin attenuates systemic immune parameters associated with T cell activation in AD *in vivo*.

### MCL-1 activity is suppressed in local and distal organ following sesamin treatment

**Figure 10:**
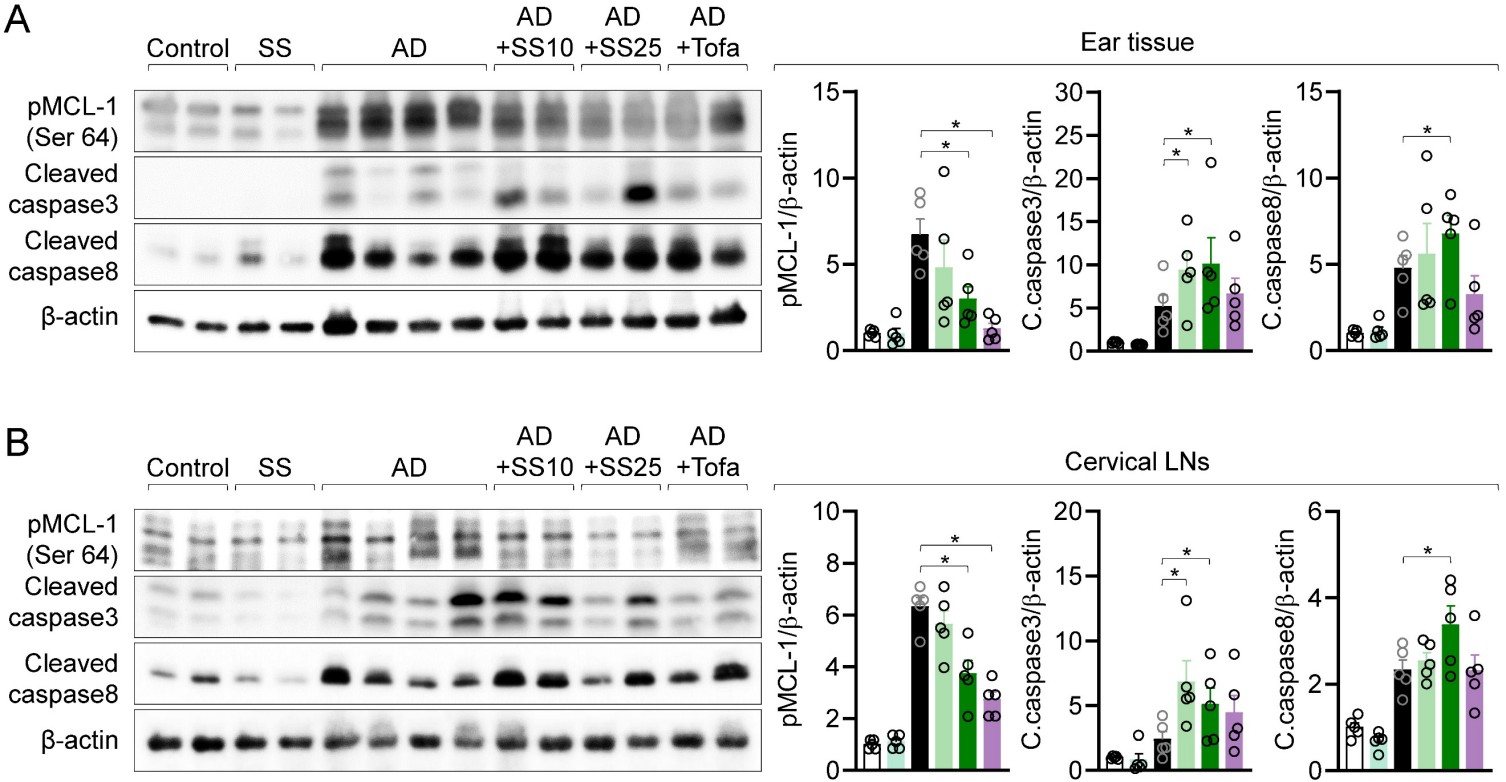
** MCL-1 activity is suppressed in local and distal organ following sesamin treatment.** (A, B) ear tissue (A) and cervical lymph nodes (B) were lysed in RIPA buffer and the expression of phosphorylated MCL-1 at Ser64, cleaved caspase3 and cleaved caspase8 were detected by Western blotting analysis. Expressions were normalized with the expression of β-actin. All results are expressed as mean ± SEM of three independent experiments. Statistical comparisons among groups were performed using one-way ANOVA with Tukey's post hoc test. A p-value less than 0.05 was considered statistically significant (*, P < 0.05).

To clarify whether the regulatory effects of sesamin on T cell activity *in vitro* are replicated *in vivo*, we measured phosphorylated MCL-1, cleaved caspase 3, and cleaved caspase 8 levels in ear tissue via western blotting. Orally administered sesamin reduced the elevated levels of phosphorylated MCL-1 in the AD model, while increasing those of cleaved caspase 3 and 8. We measured MCL-1 protein expression in cLNs to examine the systemic effects of orally administered sesamin, finding that it downregulated the elevated levels of phosphorylated MCL-1 in the AD model while upregulating those of cleaved caspase 3 and 8 (Fig. 10B). These results suggest that orally administered sesamin is associated with increased apoptotic signaling in inflamed tissues of AD mice by regulating MCL-1 activity in local tissue.

## Discussion

MCL-1 has emerged as a critical survival factor for activated and memory T cells, enabling their resistance to apoptosis and perpetuating chronic inflammation 6,7,10. In this study, sesamin was shown to directly bind to MCL-1 (Fig. 2) and inhibit its phosphorylation at Ser64, an essential post-translational modification that enhances MCL-1's stability and anti-apoptotic function (Fig. 3). Furthermore, Sepharose4B-labeled pull-down (Fig. 2C) and co-immunoprecipitation assays (co-IP, Fig. 4C) support the notion that sesamin physically interferes with the MCL-1-Bak interaction. While the current study does not include high-resolution structural analyses such as NMR or crystallography to definitively resolve the binding modality, the spatial overlap in docking models and co-IP assay strongly implies competitive inhibition.

Tissue-Resident Memory T cells (T_RM_s), particularly CD4^+^ and CD8^+^ T_RM_ subsets expressing CD69 and/or CD103, have been increasingly recognized as key drivers of disease persistence and recurrence in AD 32,33. These long-lived, non-circulating cells reside in the epidermis and dermis and maintain local cytokine production, especially IL-17 and IL-22, even in the absence of overt inflammation. Although our current study did not explicitly identify or quantify T_RM_s, the observed sesamin-induced apoptosis in activated T cells within inflamed skin raises the possibility that pathogenic T_RM_s may be indirectly affected. Given that MCL-1 is known to be essential for T_RM_ survival in peripheral tissues 34,35, we propose that sesamin's immunomodulatory effects may extend to these populations. Future investigations should evaluate the impact of sesamin on T_RM_ subsets by profiling CD69^+^CD103^+^CD49a^+^ cells via flow cytometry, distinguishing tissue-resident from circulating cells using intravascular staining, and applying single-cell transcriptomics to assess functional and apoptotic gene signatures. Additionally, adoptive transfer models and conditional *Mcl1* knockout mice could be employed to directly determine whether T_RM_ depletion contributes to the resolution of chronic inflammation 36,37. These studies would provide mechanistic insight into the therapeutic potential of sesamin as a natural agent targeting long-lived pathogenic T cell populations.

The ability of sesamin to reduce Th1/Th2/Th17 cytokine expression and serum IgE levels in an AD mouse model underscores its potential to modulate both local and systemic immune responses (Fig. 8 and Fig. 7F). Importantly, MCL-1 downregulation was observed not only in lesional skin but also in draining lymph nodes, suggesting a systemic pharmacological effect (Fig. 10). The findings align with a growing interest in targeting apoptotic regulators to selectively reshape immune responses in chronic inflammatory diseases 38. Compared to synthetic MCL-1 inhibitors currently under investigation in oncology, sesamin offers a naturally derived, orally available alternative with demonstrated safety in dietary contexts 16. In addition, our current study design focused on the treatment window during ongoing inflammation and did not include a follow-up phase to evaluate the persistence of sesamin's effects after treatment cessation. It is obvious that understanding the durability of sesamin's immunomodulatory action is highly relevant, particularly in the context of developing long-term therapies for chronic diseases such as AD. Future studies should include withdrawal and washout models to assess whether sesamin induces transient suppression or lasting immune remodeling.

This study expands the functional landscape of food-derived bioactive compounds by linking sesamin to a defined molecular target with therapeutic relevance. While prior studies have focused on its antioxidant and metabolic effects 16, our work reveals a previously unrecognized role in T cell apoptosis and immune resolution. These results provide a strong rationale for exploring sesamin and related lignans as lead compounds in the development of targeted therapies for immune-mediated diseases.

Jurkat T cells were employed in this study due to their well-established utility in modeling TCR-induced activation, proliferation, and apoptosis in human T lymphocytes. While these cells provided valuable mechanistic insights, future validation using primary human T cells, particularly those isolated from AD patient lesions or peripheral blood, would enhance the clinical applicability of our findings. Such studies would be critical to assess not only MCL-1 targeting by sesamin in human immune cells but also the variability in response due to individual immune profiles.

In addition to its role in T cells, MCL-1 is broadly expressed across multiple immune cell subsets, where it plays a critical role in maintaining cell survival. Previous studies have demonstrated that MCL-1 is essential for the development and persistence of B cells, plasma cells, eosinophils, neutrophils, and certain dendritic cell population 6,7,10,39. Given that sesamin acts as a functional MCL-1 modulator, it is plausible that its systemic administration may affect these additional cell types, especially under inflammatory conditions where MCL-1 is dynamically regulated. While our current data suggest that sesamin selectively induces apoptosis in activated T cells without significant cytotoxicity to resting cells (Fig. 5), off-target effect in other MCL-1-dependent lineages cannot be ruled out. To address this, future studies employing cell-type-specific *Mcl1* conditional knockout models and immune profiling across B cells, eosinophils, and myeloid cells would be valuable to delineate the specificity and safety of sesamin-based immunomodulation. Such investigations will help clarify whether sesamin's therapeutic window is sufficiently narrow to preserve protective immune functions while selectively eliminating pathogenic effector cells.

Although TSLP and IL-6 are well-recognized pro-inflammatory mediators in AD, particularly as early activators of keratinocyte-immune crosstalk, our data showed that their mRNA expression levels were not significantly reduced following oral administration of sesamin (supplementary Fig. 5). This suggests that sesamin may have limited direct impact on keratinocyte-driven inflammatory signaling. IL-6, in particular, plays a role in promoting Th17 polarization and sustaining chronic inflammation in the skin 40. The absence of change in IL-6 and TSLP expression implies that the anti-inflammatory effects of sesamin are unlikely to stem from broad suppression of innate immune responses or epithelial activation. Rather, our findings support a more selective mechanism in which sesamin targets activated T cells by modulating survival signaling pathways such as MCL-1-Bak interaction, leading to apoptosis of pathogenic T cells. This selective action may reduce the risk of widespread immunosuppression and preserve tissue-protective responses in keratinocytes.

Notably, our molecular docking analysis revealed that sesamin binds directly to the BH3 domain of MCL-1, specifically interacting with Arg222, a critical residue involved in the anti-apoptotic interface. This interaction likely interferes with the ability of MCL-1 to form stable heterodimers with pro-apoptotic proteins such as Bak. Supporting this mechanism, our pull-down assay demonstrated a dose-dependent reduction in MCL-1-Bak binding upon sesamin treatment. MCL-1's anti-apoptotic activity is known to rely on sequestering Bak or Bax and preventing their oligomerization and pore formation in the mitochondrial membrane. By disrupting this interaction, sesamin effectively promotes the release of Bak, leading to activation of caspase-dependent apoptotic pathways. This mechanism is consistent with our *in vivo* findings showing increased levels of cleaved caspase3/8 in inflamed skin tissues. These results strongly suggest that sesamin impairs MCL-1's survival-promoting function in activated T cells, thereby enhancing apoptosis and contributing to immune resolution. Together, this highlights the potential of MCL-1 as a therapeutic immunoregulation target and underscores sesamin's utility as a natural modulator of MCL-1-mediated signaling.

Our data further demonstrate that sesamin selectively induces in activated but not resting T cells, suggesting that its pro-apoptotic effects are activation-dependent. This selectivity appears to be mediated by differential expression of MCL-1; as shown in our study, MCL-1 is strongly upregulated in activated T cells, but not in their resting counterparts (Fig. 3A). Since sesamin binds to MCL-1 and disrupts its interaction with Bak, its apoptotic action is contingent upon the presence of MCL-1 protein. This mechanism enables sesamin to preferentially eliminate pathogenic, overactivated T cells, while sparing quiescent immune cells, which is a desirable feature for therapeutic modulation of autoimmune or hyperinflammatory conditions. These findings position sesamin as a potential natural immune checkpoint modulator targeting MCL-1-dependent survival signaling.

## Conclusion

In summary, our findings demonstrate that sesamin ameliorates the pathogenesis of AD by selectively promoting apoptosis in activated T cells through direct interaction with MCL-1. Sesamin inhibited T cell function by downregulating IL-2 production, CD69 expression, and proliferation. Mechanistically, sesamin bound to the BH3 domain of MCL-1, reduced its phosphorylation at Ser64, and disrupted MCL-1-Bak interactions, thereby enhancing caspase-dependent apoptotic signaling. Importantly, this effect was restricted to activated T cells, likely due to their elevated MCL-1 expression, highlighting sesamin's potential as a targeted immunomodulator. Further clinical studies are warranted to evaluate its utility in T cell-driven inflammatory and autoimmune diseases.

## Supplementary Material

Supplementary figures.

## Abbreviations

AD: atopic dermatitis
DNCB: 2,4-dinitrochlorobenzene
HDM extract: house dust mite extract
LC: Langerhans cell
MCL-1: myeloid cell leukemia 1
