# Supplementary material for: Sesamin Induces MCL-1-Dependent Apoptosis in Activated T Cells and Ameliorates Experimental Atopic Dermatitis
Source: Int J Biol Sci. 2025 Jul 24;21(11):4719–35. doi: 10.7150/ijbs.116753 (PMC12374809; doi:10.7150/ijbs.116753)
Supplement: Supplementary file 1 — Supplementary figures. [file ijbsv21p4719s1.pdf]

Sesamin Induces MCL-1-Dependent Apoptosis in Activated T Cells and Ameliorates Experimental Atopic Dermatitis

Hee-Suk Park[a], Woo Jung Sung[b], Yoon-Yub Park[a], Jaewoo Hong[a], Hoon-Kyu Oh[b], Hyun-Su Lee[a*]

[a]Department of Physiology, Daegu Catholic University School of Medicine, Duryugongwon-ro 17-gil 33, Daegu, Korea 42472

[b]Department of Pathology, Daegu Catholic University School of Medicine, Duryugongwon-ro 17-gil 33, Daegu, Korea 42472

***Corresponding author**

Tel.: +82-53-650-4472, E-mail: lhs6858@cu.ac.kr (HS. Lee)

***Abbreviations**

AD: atopic dermatitis, DNCB: 2,4-dinitrochlorobenzene, HDM extract: house dust mite extract, LC: Langerhans cell, MCL-1: myeloid cell leukemia 1

Supplementary figures and figure legends

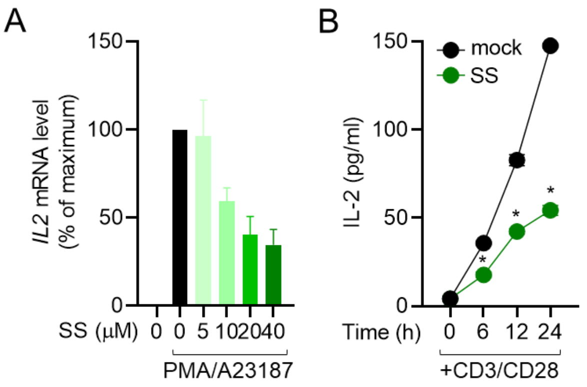

Supplementary Fig. 1. Pre-treatment with sesamin reduces IL-2 production from activated T cells

(A) Jurkat T cells (5 X 10^5/well, 12-well plate) pre-treated with indicated concentration of sesamin for 1 h were stimulated with PMA (100 nM) and A23187 (1 µM) for 6 h. Harvested cells were lysed for isolation of total RNA. The mRNA level of IL-2 gene was determined by qPCR analysis. The expression of IL-2 was normalized with the expression of GAPDH. (B) Jurkat T cells (1 X 10^4/well, 96-well plate) pre-treated with 40 µM sesamin for 1 h were stimulated with immobilized anti-CD3 (10 µg/ml) and soluble anti-CD28 (2 µg/ml) antibodies for 6 to 24 h (right). The amount of released IL-2 was evaluated by ELISA from harvested supernatants. All results are expressed as mean ± SEM of three independent experiments. Statistical comparisons among groups were performed using one-way ANOVA with Tukey's post hoc test. A p-value less than 0.05 was considered statistically significant (*, $P < 0.05$).

A

Target (Full name of protein)	Common Name	Probability
**Induced myeloid leukemia cell differentiation**	**MCL-1**	**0.78296**
Hypoxia-inducible factor 1 alpha	HIF1A	0.10934
Arachidonate 5-lipoxygenase	ALOX5	0.10934

B

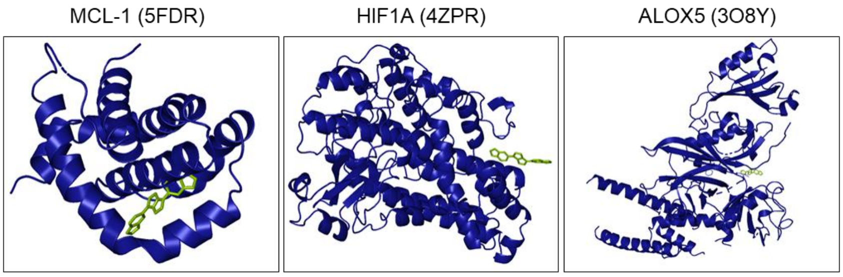

C

Target (Common name of protein)	Affinity Energy	Ki value	Ligand Efficiency
**MCL-1**	**10.2 kcal/mol**	**33.37 nM**	**-0.39**
HIF1A	6.5 kcal/mol	17.2 μM	-0.25
ALOX5	6.3 kcal/mol	24.1 μM	-0.24

Supplementary Fig. 2. Prediction results and molecular docking analysis of top three candidates predicted to bind to sesamin

(A) Top three target candidates of sesamin in human and mouse T cells were predicted by swisstargetprediction website after cut off below 0.1 and listed according to the probability. (B) Molecular docking analysis of top three target candidates with sesamin generated by AMdock and pyMOL software. (C) Affinity energy, KI value and ligand efficiency of top three target candidates predicted to bind to sesamin were obtained from AMdock and pyMOL software.

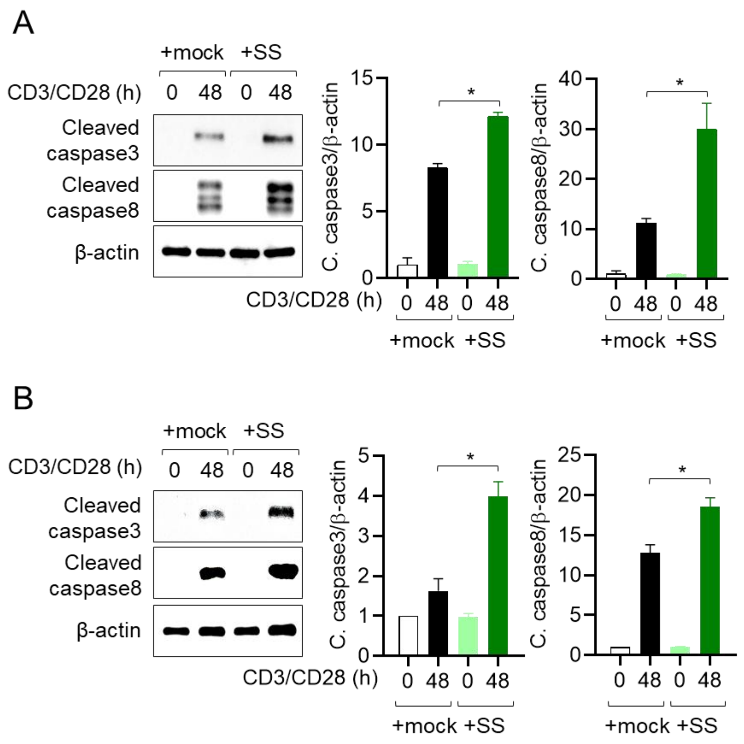

Supplementary Fig. 3. Sesamin promotes cleavages of caspase3 and caspase8 in activated T cells

(A, B) Jurkat T cells (1 X 10^6/well, 12-well plate, A) or mouse $CD4^+$ T cells (3 X 10^6/well, 12-well plate, B) pre-treated with 40 μM sesamin for 1 h were stimulated with immobilized anti-CD3 (10 μg/ml) and soluble anti-CD28 (2 μg/ml) antibodies for 48 h. Harvested cells were lysed in RIPA buffer and the expression of cleaved caspase3 and caspase8 were detected by Western blotting. Expressions were normalized with the expression of β-actin. Expressions were normalized with the expression of β-actin. All results are expressed as mean ± SEM of three independent experiments. Statistical comparisons among groups were performed using one-way ANOVA with Tukey's post hoc test. A p-value less than 0.05 was considered statistically significant (*, $P < 0.05$).

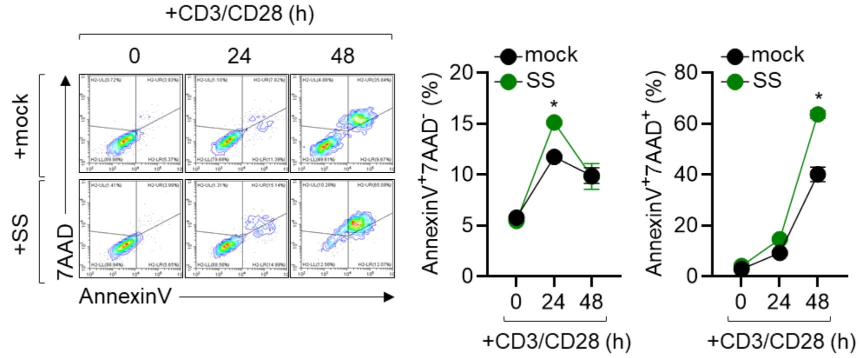

Supplementary Fig. 4. Sesamin enhances apoptotic populations exclusively in activated T cells

Jurkat T cells (5 X 10^5/well, 12-well plate) pre-treated with 40 μM sesamin for 1 h were stimulated with immobilized anti-CD3 (10 μg/ml) and soluble anti-CD28 (2 μg/ml) antibodies for 24 to 48 h. Harvested cells were stained with AnnexinV and 7AAD for AnnexinV apoptosis assay. From Contour Plot, the population of AnnexinV$^+$7AAD$^-$ and AnnexinV$^+$7AAD$^+$ was analyzed. All results are expressed as mean ± SEM of three independent experiments. Statistical comparisons among groups were performed using one-way ANOVA with Tukey's post hoc test. A p-value less than 0.05 was considered statistically significant (*, $P < 0.05$).

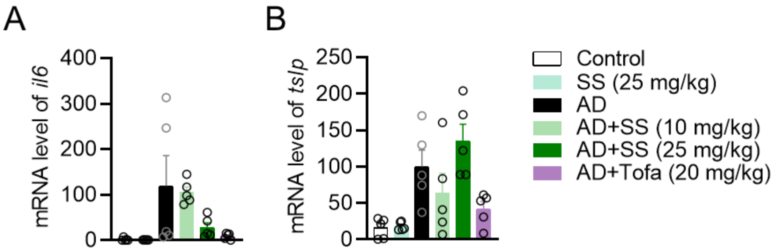

Supplementary Fig. 5. Oral administration of sesamin does not reduce the mRNA level of IL-6 and TSLP in the ear of mice with HDM extract/DNCB-induced AD

(A, B) mRNA levels of *il6* (A) and *tslp* produced from keratinocytes (B) from ear tissues of each mouse. The expression of target genes was normalized with the expression of *Gapdh*. All results are expressed as mean ± SEM of three independent experiments. Statistical comparisons among groups were performed using one-way ANOVA with Tukey's post hoc test. A p-value less than 0.05 was considered statistically significant (*, $P < 0.05$).
